# Sterol interactions influence the function of Wsc sensors

**DOI:** 10.1016/j.jlr.2023.100466

**Published:** 2023-11-02

**Authors:** Lukas Bernauer, Paula Berzak, Leonie Lehmayer, Julia Messenlehner, Gustav Oberdorfer, Günther Zellnig, Heimo Wolinski, Christoph Augustin, Melanie Baeck, Anita Emmerstorfer-Augustin

**Affiliations:** 1 Institute of Molecular Biotechnology, Graz University of Technology, NAWI Graz, Graz, Austria; 2 BioTechMed-Graz, Graz, Austria; 3 Institute of Biochemistry, Graz University of Technology, NAWI Graz, Graz, Austria; 4 Institute of Biology, Plant Sciences, University of Graz, NAWI Graz, Graz, Austria; 5 Department of Molecular Biosciences, University of Graz, NAWI Graz, Graz, Austria; 6 Gottfried Schatz Research Center, Medical University Graz, Graz, Austria; 7 Austrian Centre of Industrial Biotechnology, acib GmbH, Graz, Austria

**Keywords:** Cell wall integrity, *Komagataella phaffii*, Wsc proteins, sterol-binding motif, MAPK signaling

## Abstract

The Wsc1, Wsc2, and Wsc3 proteins are essential cell surface sensors that respond to cell wall perturbation by activating the cell wall integrity pathway (CWIP). We show here that in situ production of cholesterol (in place of ergosterol) induces hyper-phosphorylation of Slt2, the MAPK of the CWIP, and upregulates cell wall biosynthesis. Deletion of all three Wsc genes in *K. phaffii* reverts these phenotypes. In the cholesterol-producing strain, both Wsc1 and Wsc3 accumulate in the plasma membrane. Close inspection of the transmembrane domains of all three Wsc proteins predicted by AlphaFold2 revealed the presence of CRAC sterol**-**binding motifs. Experiments using a photoreactive cholesterol derivative indicate intimate interaction of this sterol with the Wsc transmembrane domain, and this apparent sterol binding was abrogated in Wsc mutants with substitutions in the CRAC motif. We also observed cholesterol interaction with CRAC-like motifs in the transmembrane domains of mammalian integrins, analogs of Wsc proteins. Our results suggest that proper signaling of the Wsc sensors requires highly specific binding of the native endogenous terminal sterol, ergosterol.

Cell walls are essential for the survival of fungal cells and stresses or damage to the cell wall activate a response known as the cell wall integrity pathway (CWIP). At the top of the CWIP are two classes of sensors, Wsc proteins and Mid proteins, which serve as the initial detectors of cell wall perturbation (reviewed by (1)). The types of stimuli that evoke the CWIP are manifold, including growth at elevated temperatures (2), treatment with the glucan synthase inhibitor caspofungin or the chitin-binding dye calcofluor white (3, 4), and, for methyltrophic yeasts, like *Komagataella phaffii* (formerly *Pichia pastoris*), even upon a shift from glucose to methanol as the carbon source (5). Activation of the CWIP sensors stimulates the action of two guanine nucleotide exchange factors Rom1 and Rom2 (6), whose substrate is the small GTPase Rho1. The Rho1-GTP generated binds to and activates the protein kinase Pkc1. Pkc1, in turn, activates a classical three-tiered MAPK cascade, resulting in phosphorylation and activation of Slt2 (also known as Mpk1), the terminal MAPK of the CWIP. Activated Slt2 enters the nucleus, where it induces the transcription of at least 25 genes, most of which encode cell wall proteins or have been otherwise implicated in cell wall biogenesis (7).

At the structural level, and focusing on the Wsc sensors, the three proteins encoded by the *K. phaffii* genome have similar overall structures (Fig. 1). They possess a Cys-rich N-terminal fold (∼110 residues), including a characteristic PAN/Apple domain that mediates protein–protein and protein–carbohydrate interactions (6), followed by a long (∼150 residues) Ser/Thr-rich "stalk" that is highly O-mannosylated (8), a modification believed to confer rigidity to the proteins that allows them to act as mechanosensors (9), and, thereafter, a single, highly hydrophobic, α-helical transmembrane segment trailed by a cytosolic tail of significant length (∼80–100 residues) that contains signal sequences for endocytosis (10, 11) and interacts with the downstream effectors needed to elicit the MAPK pathway (9). It is thought that changes in cell wall architecture cause corresponding conformational changes in the extracellular domains of the Wsc proteins, which then are transduced across the membrane to these intracellular effectors (reviewed by (12)). Although much is already known about the different domains of the CWIP sensors, the exact molecular mechanism by which stress is transmitted from the extracellular portions to the intracellular signaling apparatus, especially the role of the transmembrane domain, remains an open question.

**Fig. 1:**
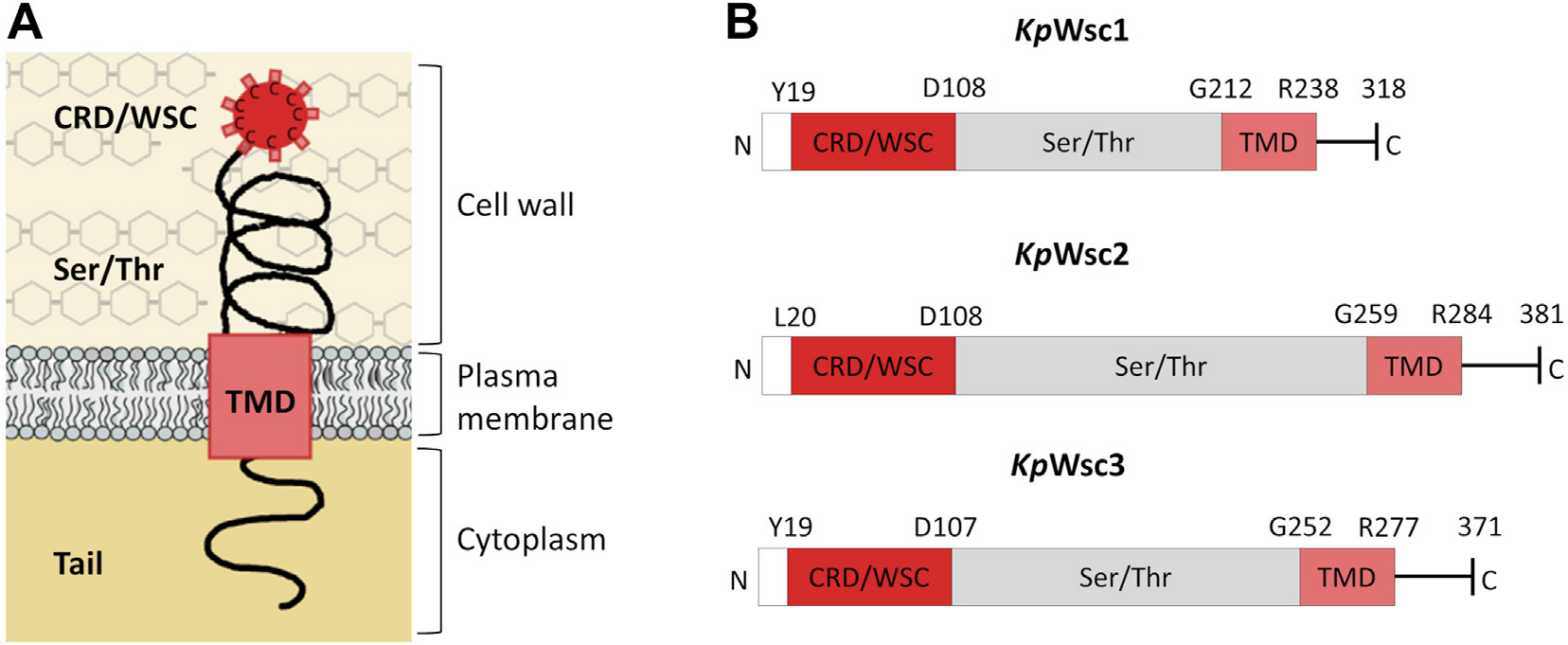
Architecture and structural model of *K. phaffii* Wsc-type sensors. A: Model showing the topology and proposed functional domains of Wsc-type sensors, including the N-terminal cysteine-rich domain (CRD) or WSC domain, the glycosylated serine/threonine-rich domain (Ser/Thr), the transmembrane domain (TMD), and the C-terminal cytoplasmic tail. B: Domain architecture of the *K. phaffii* Wsc family mechanosensors Wsc1 (protein ID NP_014650.1), Wsc2 (protein ID CCA37335.1), and Wsc3 (protein ID CCA37334.1). Residues were assigned based on a ClustalW multiple sequence alignment with *Sc*Wsc1 (UniProt entry P54867), *Sc*Wsc2 (UniProt entry P53832), and *Sc*Wsc3 (UniProt entry Q12215).

Several studies have reported links between the CWIP and biosynthesis of the fungal-specific sterol ergosterol. For example, knockout of individual Wsc proteins in *Saccharomyces cerevisiae* confers greater susceptibility to fluconazole, an antifungal agent that inhibits a P-450 enzyme (C-14 α-demethylase) required for ergosterol synthesis (13, 14). Another study (15) reported that, in *Cryptococcus neoformans*, the anti-fungal action of caspofungin synergizes with fluconazole in combination with amphotericin B, an antibiotic that binds ergosterol and causes cell death by pore formation (16). A more recent study found that a loss-of-function mutation in *ERG9*, an early essential gene of the ergosterol biosynthesis, strongly exacerbated the cell wall integrity defect of cells deficient in the synthesis of the complex sphingolipid MIPC and, conversely, upregulation of ergosterol biosynthesis ameliorated the cell wall defect (17).

The CWIP is highly conserved among fungi (18). The Wsc- and Mid-type sensors have been investigated thoroughly in *S*. *cerevisiae* and, to some extent, in fission yeast (19) and *K. phaffii* (5, 20). *S. cerevisiae* harbors three Wsc-type and two Mid-type sensors (reviewed by (21)), whereas *K. phaffii* contains three Wsc-type sensors (Wsc1, Wsc2, and its paralogue Wsc3) (5), but no orthologues of Mid-type sensors (20). The lack of any Mid-type sensors makes *K. phaffii* a convenient organism in which to study the function of Wsc-type sensors. In this regard, we previously demonstrated that exchange of non-native cholesterol for endogenous ergosterol in *K. phaffii* results in hyper-stimulation of cell wall biosynthesis (22).

To understand how cholesterol production elevates cell wall biosynthesis in *K. phaffii*, a reasonable target of this change in sterol could be one or more of the Wsc sensors because they are embedded in the plasma membrane where the bulk of any sterol resides. In fact, aside from their roles in controlling membrane fluidity, permeability, and rigidity (23, 24), it has been demonstrated that sterols often interact with transmembrane proteins and these interactions are important in the function, localization, and stability of the interacting proteins (25). In most cases, sterol binding is mediated by a sequence element termed a CRAC (cholesterol recognition amino acid consensus) motif with the pattern –L/V–X_(1–5)_–Y–X_(1–5)_–R/K– (26) and generally located in a portion of the protein in contact with the inner (cytosolic) leaflet of the membrane (and its mirror sequence, dubbed a CARC motif, generally located in a portion of the protein in contact with the outer (exocellular) leaflet of the membrane). Because the statistical probability of finding such a low-complexity sequence is rather high, the mere presence of a putative CRAC or CARC motif does not suffice as proof of specific protein-sterol interaction.

Given our finding that cholesterol-producing *K. phaffii* exhibit elevated cell wall biosynthesis, we first examined whether the transmembrane segments any of the Wsc proteins contain potential CRAC motifs. Finding candidates, we then explored methods to determine if they represent authentic sterol-binding elements and we were able to detect such an interaction. Based on the data presented here, it seems that the modest structural differences between cholesterol and ergosterol are sufficient to explain why the CWIP is upregulated in the cholesterol-producing cell, suggesting further that ergosterol is needed to maintain proper Wsc sensors signaling.

## Materials and Methods

### Cloning and strain construction

All plasmids used in this study were generated using Gibson assembly (27). pPpHyg-Cas9 plasmids were adapted from Lehmayer *et al.*, 2022 (20). The sgRNA targeting sequence was adapted to target *SLT2* for HA-tagging, the *his4*Δ locus for the insertion of diverse expression cassettes, or *WSC1* for gene deletion (supplemental Table S1). Repair and insertion plasmids were constructed from pPpKC2 and pPpKC3 (28). Repair cassettes were cut out of readily cloned vectors using *Smi*I and bands of the correct size were gel purified (supplemental Table S1). All PCRs were performed using Phusion™ DNA polymerase (Thermo Fisher Scientific Inc., St. Leon-Rot, Germany), and the fidelity of all constructs was verified by nucleotide sequence analysis. Maps of plasmids used for strain generation are available as Expanded View Files.

Transformation of *K. phaffii* was done according to the condensed protocol of Lin-Cereghino *et al.* (29). For conventional, homologous recombination using purified cassettes and selection pressure, cells were transformed with ca. 500 ng of the respective DNA fragment and plated onto minimal dextrose (MD) media [2% glucose, 13.4 g L^−1^ Yeast Nitrogen Base (without amino acids), 4∗10 5% biotin] lacking histidine. For gene editing using CRISPR/Cas9 we followed the protocol of Weninger at al., 2016 (30). In short, cells were co-transformed with 100 ng of pPpHyg-Cas9 plasmid and 500 ng of the respective repair cassette (supplemental Table S1), and plated onto YPD plates containing 200 μg/ml hygromycin. Correct integration of cassettes into the yeast genome was verified by cPCR and sequencing. For gene knock-out via CRISPR/Cas9 without a repair cassette, cells were simply transformed with 100 ng of pPpHyg-Cas9 targeting the respective gene (supplemental Table S1). Correct integration of expression cassettes into the yeast genome was conﬁrmed by colony PCR and sequencing. All strains used in this study are summarized in supplemental Table S2.

### Cultivations of cells

All *K. phaffii* strains used in this study were grown on minimal dextrose (MD) media [2% glucose, 13.4 g L^−1^ Yeast Nitrogen Base (without amino acids), 4∗10 5% biotin, 0.4% histidine to permit growth of auxotrophs]. *S. cerevisiae* strains were grown on defined minimal (SD) media [2% glucose, 6.7 g L^−1^ Yeast Nitrogen Base (without amino acids), 400 μM adenine, 400 μM tyrosine, 400 μM tryptophane, 300 μM histidine, 200 μM uracil, 1 mM lysine and 2 mM leucine]. Cultures were propagated at 28°C and harvested at indicated time points.

For the preparation of glycerol stocks, 5 ml of YPD [1% yeast extract, 2% peptone, 2% glucose] were inoculated with cells, incubated in a shaker at 28°C overnight, pelleted at 5,000 rpm for 5 min, resuspended in 0.5 ml of YPD and 0.5 ml of 50% glycerol and frozen at −80°C.

### Electron microscopy imaging and quantification

Cells were cultivated at 28°C with shaking at 170 rpm in baffled flasks using MD+his medium until they reached the early exponential phase. Cells were pelleted, washed with 15 ml of ddH_2_O, pelleted again, and fixed for 5 min in 5 ml of a 1% aqueous solution of KMnO_4_ at room temperature. Next, cells were washed with 10 ml of ddH_2_O, and fixed by adding 5 ml of a 1% aqueous solution of KMnO_4_ for 20 min. Fixed cells were washed four times in 10 ml of ddH_2_O for 10 min and incubated in 0.5% aqueous uranyl acetate overnight at 4 °C. Samples were then dehydrated in a graded series of 50%, 70%, 90%, and 100% ethanol for 20 min each. Pure ethanol was then exchanged with a mixture of propylene oxide and ethanol (1:1) for 10 min and then finally with pure propylene oxide for 10 min. Specimens were gradually infiltrated with increasing concentrations (30%, 50%, 70%, and 100%) of Agar 100 epoxy resin mixed with propylene oxide for a minimum of 3 h per step. Samples were embedded in pure, fresh Agar 100 epoxy resin and polymerized at 60 °C for 48 h. Ultrathin sections of 80 nm were stained for 3 min with lead citrate and viewed with a Zeiss Libra120 Plus transmission electron microscope (Carl Zeiss AG, Oberkochen, Germany).

### Spot assays

*K. phaffii* strains were cultivated in BMD buffered with 100 mM K_2_HPO_4_/KH_2_PO_4_ (pH 6) overnight until they reached saturation. Cells were then diluted to an OD_600_ of one, and five-fold serial dilutions were prepared in sterile 96-well plates. Samples of each dilution were spotted, using a Steers-type multipronged inoculator, onto agar plates containing phosphate-buffered BMD medium and either 1 mM 1-NAA (Sigma Aldrich) in DMSO or an equivalent volume of DMSO (vehicle) (Lehmayer *et al.*, 2022). Plates were incubated at 28°C and typically photographed after 72 h.

### Immunoblot analysis

**Fig. 2:**
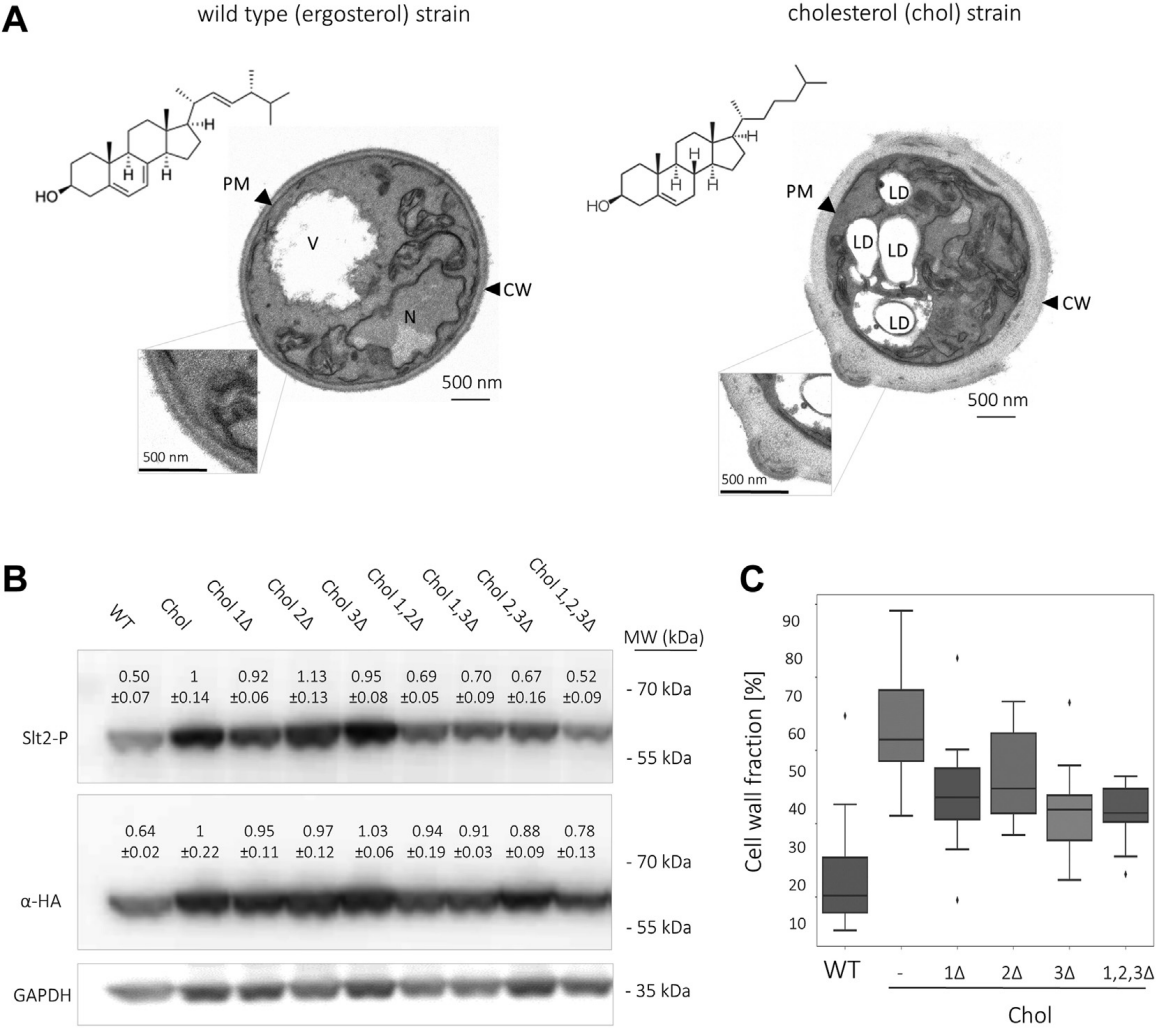
Wsc proteins upregulate cell wall biosynthesis when ergosterol is exchanged for cholesterol in *K. phaffii*. A: The *K. phaffii* CBS7435 *his4*Δ (WT) strain, and an isogenic derivative harboring *erg5*::DHRC7 and *erg6*::DHCR24 gene insertions for cholesterol production (MH458; chol) were cultivated to early exponential phase and imaged by electron microscopy as described in Materials and Methods. Scale bar, 500 nm. PM: plasma membrane, CW: cell wall, V: vacuole, N: nucleus, LD: lipid droplets. B: A CBS7435 *his4*Δ strain expressing *SLT2-HA* from the endogenous *SLT2* locus (yAEA361), a cholesterol-producing strain expressing *SLT2-HA* from the endogenous *SLT2* locus (yAEA386), and isogenic derivatives of yAEA386 carrying deletions of *wsc1*Δ (yAEA366), *wsc2*Δ (yAEA387), *wsc3*Δ (yAEA367), *wsc1*Δ *wsc2*Δ (yAEA390), *wsc1*Δ *wsc3*Δ (yAEA377), *wsc2-3*Δ (yAEA369), and *wsc1*Δ *wsc2*Δ *wsc3*Δ (yAEA379) were cultivated to middle exponential phase at 28°C, harvested, lysed, and proteins were extracted, resolved by SDS–PAGE, and analyzed by immunoblotting with Phospho-p44/42 MAPK (Erk1/2) (Thr202/Tyr204) antibody and anti-HA antibody, as described under Materials and Methods. Loading control, GAPDH detected on the same immunoblots using anti-GAPDH antibody. MW, marker proteins (kDa). Values above the lanes represent the percentage of relative protein levels (average of three independent experiments with SEM). C: The same strains as in B (except for double knockout strains) were grown to the middle exponential phase and imaged by TEM. Cell wall areas from electron microscopy images (n = 15 per strain) were quantified using a “Denoising and Segmentation Tool” as described in Materials and Methods, and results were plotted in box-and-whisker format.

All steps were performed at 4°C. The cells in samples (1 ml) of an exponentially growing culture (A600 nm = 4) were collected by brief centrifugation, lysed by resuspension in 300 μl of 1.85 M NaOH, 7.4% β-mercaptoethanol. Protein in the resulting lysate was precipitated by the addition of 300 μl of 50% trichloroacetic acid (TCA) for 1 h. Afterward, the samples were centrifuged for 10 min at 10,000 rpm. After discarding the supernatant, the pellets were washed with 1 ml of ice cold ddH_2_O and resuspended in 50 μl of sample buffer (For 100 μl of sample buffer, 16.5 μl of NuPAGE™ LDS Sample Buffer, 33 μl of 1 M Tris-Base, 2 μl of β-mercaptoethanol and 49.5 μl of ddH_2_O were mixed.). Samples were briefly centrifuged at 10,000 rpm for 20 s, and 12 μl of the supernatant resolved by NuPAGE™ Mini Protein Gels (12%, Bis-Tris, 1.0 mm; Thermo Fisher Scientific Inc) at 200 V. Resolved proteins were transferred electrophoretically to a nitrocellulose membrane using a wet transfer apparatus (NuPAGE™, Thermo Fisher Scientific Inc). For blocking of unspecific interactions, the membranes were incubated for 1 h in TBST-BSA (5%). After blocking, the membranes were incubated overnight at 4°C in TBST-BSA (2.5%) with an appropriate primary antibody (at the indicated dilution): mouse ANTI-FLAG® M2-Peroxidase (HRP) antibody (1:4,000; Sigma Aldrich); peroxidase-conjugated anti-HA 3F10 from rat (1:2,500; Roche); Phospho-p44/42 MAPK (Erk1/2) (Thr202/Tyr204) antibody (1:500; Cell Signaling Technology) and rabbit anti-GAPDH (1:5,000; Institute of Biochemistry, Graz University of Technology, Austria) (31). In *S. cerevisiae*, a Slt2-specific antibody exists (anti-Mpk1, catalog No. sc-6803; Santa Cruz), which was specifically raised against amino acids 453–491 close to the C-terminus of *Sc*Slt2. Unfortunately, *Sc*Slt2 and *Kp*Slt2 exhibit very low sequence homology in this specific region, resulting in poor affinity of this antibody for *Kp*Slt2. Following a strategy published for *Sc*Slt2-HA (32), HA-tagged variants of Slt2 were generated by inserting an HA-tag at position I409, using a CRISPR/Cas9 approach (supplemental Table S1), in order to compare phosphorylation states to overall Slt2 protein levels. Since strains expressing HA-tagged versions of Slt2 grew slower than the respective wild-type strains, we used them exclusively for the immunoblot shown in Fig. 2 and for no other experiments where functional CWI-pathway was required. After washing with TBST (three times with ≥10 ml), membranes were either used directly for immunodetection, or incubated with an appropriate HRP-conjugated secondary antibody - goat Anti-Rabbit IgG–Peroxidase antibody A9169 (1:10,000, Sigma Aldrich), and then washed with TBST (three times with ≥10 ml). Enhanced chemiluminescent signal detection (Clarity Max Western ECL Substrate, Bio-Rad) was used to visualize immunoreactive bands. Molecular weight marker used was the PageRuler™ pre-stained protein ladder (Thermo Scientific™). Band intensities were quantified using the ROI manager tool from Fiji and normalized using the sample-specific GAPDH signal (33).

### Fluorescence microscopy

Cells were diluted to an OD_600_ of 0.1 and grown in a minimal medium containing histidine until they reached an OD_600_ of 2. Microscopy was performed on a Leica SP8 confocal microscope (Leica Microsystems Inc., Germany) and using a 63×, NA 1.4 HCX PL APO oil immersion objective. mNeonGreen was excited at 488 nm and emission was detected between 500-550 nm. Fluorescence and transmission images were acquired simultaneously. Images shown in one Figure were taken the same day to avoid differences in laser intensity.

### Cell segmentation

For quantifications of cell wall areas, cells were imaged by electron microscopy. Images taken from mid-sections of similar-sized cells were processed using a “Denoising and Segmentation Tool” (34), which enabled efficient and reliable segmentation of cell wall areas. Black and white images generated by this software were evaluated using Python, version 3.11.2, and the OpenCV library (35), version 4.6.0. Cell wall area was normalized to total cell area. Data from at least 15 images per strain were used in Box Blots.

### Photoclick assay

The interaction assay between a clickable, photoreactive sterol probe (PhotoClick Cholesterol) and TMD-mNG fusions was done as described previously, with minor adjustments (Chauhan *et al.*, 2020). Briefly, 500 ml of cells were grown to the middle exponential phase, harvested by centrifugation (5,000 rpm at 4°C for 5 min), washed with ddH_2_O, and centrifuged again. The cell pellet was resuspended in 25 ml of 20 mM TE buffer (pH 8.0) containing 1 μM PMSF and disrupted using glass beads. Next, cell debris was pelleted at 5.000 rpm for 10 min. The supernatant was centrifuged again at 10,000 for 10 min and the resulting supernatant ultracentrifuged for 45 min at 4°C and 45,000 rpm. The resulting microsomal fractions were resuspended in 1 ml of PBS buffer and 175 μl thereof were incubated with 25 μl of a 120 μM PhotoClick Cholesterol (Avanti Polar Lipids, Inc.) stock solution for 1 h at room temperature. The samples were distributed equally in four wells (50 μl per well) of a 96-well plate and irradiated with 365 nm longwave UV light on ice for 20 min. After irradiation, the aliquots were recombined.

Dde Azide Agarose (Click Chemistry Tools; 80 μl per 200-μl sample to be processed) was washed three times with 1 ml of PBS and resuspended in 160-μl PBS + 0.5% SDS. For preparation of the Click Chemistry Master Mix, 60 μl of tris[(1-benzyl-1H-1,2,3-triazol-4-yl)methyl]amine (TBTA, TCI Chemicals; 1.7 mM in 1:4 v/v DMSO:tert-butanol), 20 μl of 50 mM CuSO_4_ (freshly prepared), and 20 μl of 50 mM tris(2-carboxyethyl)phosphine hydrochloride (TCEP, Sigma; freshly prepared) were combined. In all, 200 μl of cross-linked PhotoClick Cholesterol samples were mixed with 160 μl of Dde Azide Agarose suspensions, and the click reaction was initiated by adding 40 μl of the Master Mix. Samples were vortexed and incubated in an end-over-end shaker for 3 h at room temperature. Dde Azide Agarose was pelleted by centrifugation at 2,500 rpm for 1 min at 4°C. After removal of the supernatant, agarose beads were washed with 100 mM Tris/HCl (pH 7.5), 1% SDS, 250 mM NaCl, and 5 mM EDTA five times. Sterol-protein complexes were eluted from the beads by incubation with 2% (v/v) hydrazine (Sigma) in PBS for 2 h at room temperature. Samples were centrifuged at 2,500 rpm for 1 min at 4°C. 12 μl of the resulting supernatant were mixed with 3 μl of NuPAGE™ sample buffer (16.5 μl of NuPAGE™ LDS Sample Buffer, 33 μl of 1 M Tris-Base, 2 μl of β-mercaptoethanol and 49.5 μl of ddH_2_O) and loaded onto a NuPAGE™ Mini Protein Gel (Thermo Fisher) for a immunoblot analysis.

### Alpha-fold predictions

Structures of the Wsc1 and Wsc3 transmembrane helices (GGSNRGALIGGAVGGVVGALIIFSLAFFFTWRRIHNNKSD; LSKGAIAGTVIGSVIGGVLIIVALAFWWWRRRKSDIESDL) were predicted using AlphaFold2 (36). The Cholesterol ligand was built with Avogadro2 (37) and geometry optimized using the MMFF94 force field. Partial charges for the ligand were computed with Chimera (38) using the semiempircal AM1-BCC method. A pdb file was generated from the Chimera output mol2 file, using a python script (molfile_to_params.py) included in the Rosetta software suite (39) to get subsequent labeling of the atoms. Ligand docking was performed by uploading all necessary files in pdb format to the HADDOCK2.4 server (40, 41). Docking parameters were adjusted to enable sampling of the ligand around F273, which was shown experimentally to interact with the ligand. During the docking runs the following sampling parameters were set: number of rigid body docking structures: 10,000; number of structures for semi flexible refinement: 400; number of structures for final refinement 400. The resulting docked structures were analyzed by energy, sampling density and plausible ligand orientations. To visualize the membrane orientation of the peptides, the OPM server (42) was used.

## Results

### Wsc proteins upregulate the CWI pathway in cholesterol producing yeasts

We recently discovered that a cholesterol-producing *K. phaffii* strain produces ∼4 times thicker cell walls (22). To confirm and expand these results, we imaged wild type and cholesterol-producing (chol) yeast cells by transmission electron microscopy (TEM). In addition to *K. phaffii* (Fig. 2A), we also investigated *S. cerevisiae* strains (supplemental Fig. S1A) and observed similar effects. Cholesterol production also leads to enlarged cell wall structures in *S. cerevisiae*, but the effect was less pronounced than in *K. phaffii*. Next, we examined Slt2 phosphorylation using an anti-Slt2-P/phospho-p42/44 MAPK-specific antibody. In the *K. phaffii* (Fig. 2B), and to some extent in the *S. cerevisiae* cholesterol strain (supplemental Fig. S1B), Slt2 production and phoshorylation were significantly upregulated. To investigate, whether Wsc proteins respond to cholesterol production by stimulating production and hyperphosphorylation of Slt2, and consequently, upregulating cell wall biosynthesis, we deleted all three *WSC* genes in *K. phaffii*. In the deletion strains, Slt2 and p-Slt2 levels dropped, with the lowest levels observed for the *wsc1*Δ *wsc2*Δ *wsc3*Δ strain (Fig. 2B). Quantifications of the cell wall areas in TEM images using a ‘Denoising and Segmentation Tool’ (34) and the OpenCV library to evaluate the resulting black/white images (supplemental Fig. S2A) showed that the enlarged cell wall thickness also decreases in the absence of Wsc proteins in *K. phaffii* (Fig. 2C). As a control, we checked whether the loss of Wsc proteins could be complemented by reintroducing single copies of *WSC* genes in the *wsc1*Δ *wsc2*Δ *wsc3*Δ triple deletion strain. While Slt2 levels were almost back to wild type when *WSC1* and *WSC3* were re-expressed, the hyperproduction of cell walls only recovered partially (supplemental Fig. S2C).

### Wsc proteins accumulate in the cholesterol producing *K. phaffii* strain

**Fig. 3:**
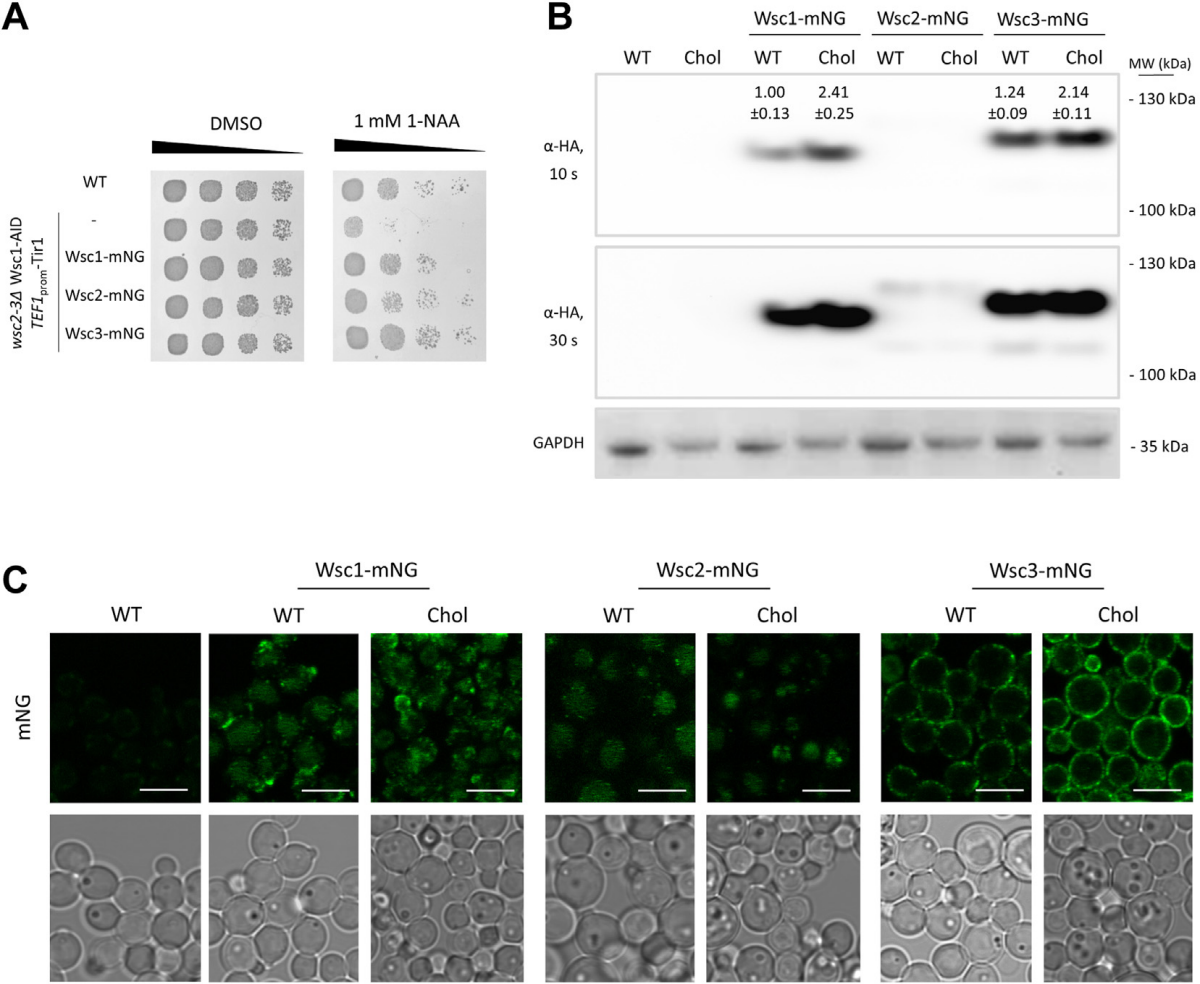
Cholesterol production causes accumulation of Wsc1 and Wsc3 in the plasma membrane. A: Functional expression of mNG-tagged Wsc proteins was verified by a complementation assay. For this, the *K. phaffii* strain CBS7435 *his4*Δ, a *wsc2-3*Δ double mutant derivative expressing *WSC1*-AID∗ as well as OsTIR1 from the *TEF1* promoter (yLL142), or otherwise isogenic strains expressing *WSC1*-mNG (yLB240), *WSC2*-mNG (yLB241) or *WSC3*-mNG (yLB243) from their endogenous promoters from the *his4*Δ locus were grown in minimal media to late exponential phase, and spotted either on vehicle plates containing 1% DMSO or on plates containing 1 mM 1-NAA as described in Materials and Methods. B: To compare expression levels of mNG-tagged *WSC* genes, a *K. phaffii* wild type (CBS7435 *his4*Δ) and cholesterol (MH458) strain and both strains expressing *WSC1*-mNG (yAEA345 and yAEA348), *WSC2*-mNG (yLB130 and yLB136) and *WSC3*-mNG (yLB133 and yLB139) from their endogenous promoters in the *his4Δ* locus were grown at 28°C to middle exponential phase, harvested, lysed, and proteins were extracted, resolved by SDS–PAGE, and analyzed by immunoblotting with anti-HA antibody, as described under Materials and Methods. Loading control, GAPDH detected on the same immunoblots using anti-GAPDH antibody. MW, marker proteins (kDa). Values above the lanes represent the percentage of relative protein levels (average of three independent experiments with SEM). C: The same strains as in B were examined by fluorescence microscopy and representative images are shown. Scale bar, 5 μm.

To investigate the localization of Wsc1, Wsc2 and Wsc3 in *K. phaffii*, N-terminal mNeonGreen(mNG)- fusions were generated. First, growth complementation assays were performed by applying the auxin-inducible degron system to confirm the functional expression of mNG-tagged *WSC* genes (Fig. 3A). As published by our group, auxin-induced degradation of an AID-tagged Wsc1 leads to severe growth defects in a *wsc2*Δ *wsc3*Δ double knockout strain (20). We reintroduced copies of either *WSC1*-mNG, *Wsc2*-mNG, or *Wsc3*-mNG into this strain and checked the growth of these cells on plates containing 1-NAA, a synthetic auxin analog. Wsc1-mNG and Wsc3-mNG could fully, and Wsc2-mNG partially rescue the growth defect observed in a *wsc2-3*Δ Wsc1-AID∗ strain, when Wsc1-AID∗ was degraded by the auxin-inducible degron system. Immunoblot analysis of wild-type and cholesterol strains confirmed the expression of all three *WSC*-mNG fusion genes. *WSC2* seemed to be barely expressed, and faint bands only became visible when membranes were overexposed (Fig. 3B). In cholesterol-producing strains, higher amounts of Wsc1 and Wsc3 were found. Next, cells were observed by fluorescence microscopy. In general, Wsc1, Wsc2, and Wsc3 showed very similar localizations than reported for the respective homologous proteins in *S. cerevisiae* (14, 43). Wsc1 was enriched in single, small dots in the plasma membrane, especially at sites of polarized growth (Fig. 3B). Signals for Wsc2 were very faint and only a small number of dots became visible at the plasma membrane. Also, some signal was detected in the vacuole, which may be an indication of protein degradation. Wsc3 was evenly distributed all over the plasma membrane. In cholesterol-producing strains, Wsc1 and Wsc3 were enriched in the plasma membranes, confirming the increased amounts found in immunoblots.

### CRAC-motif amino acids are present in Wsc protein transmembrane domains and are required for sterol interactions

**Fig. 4:**
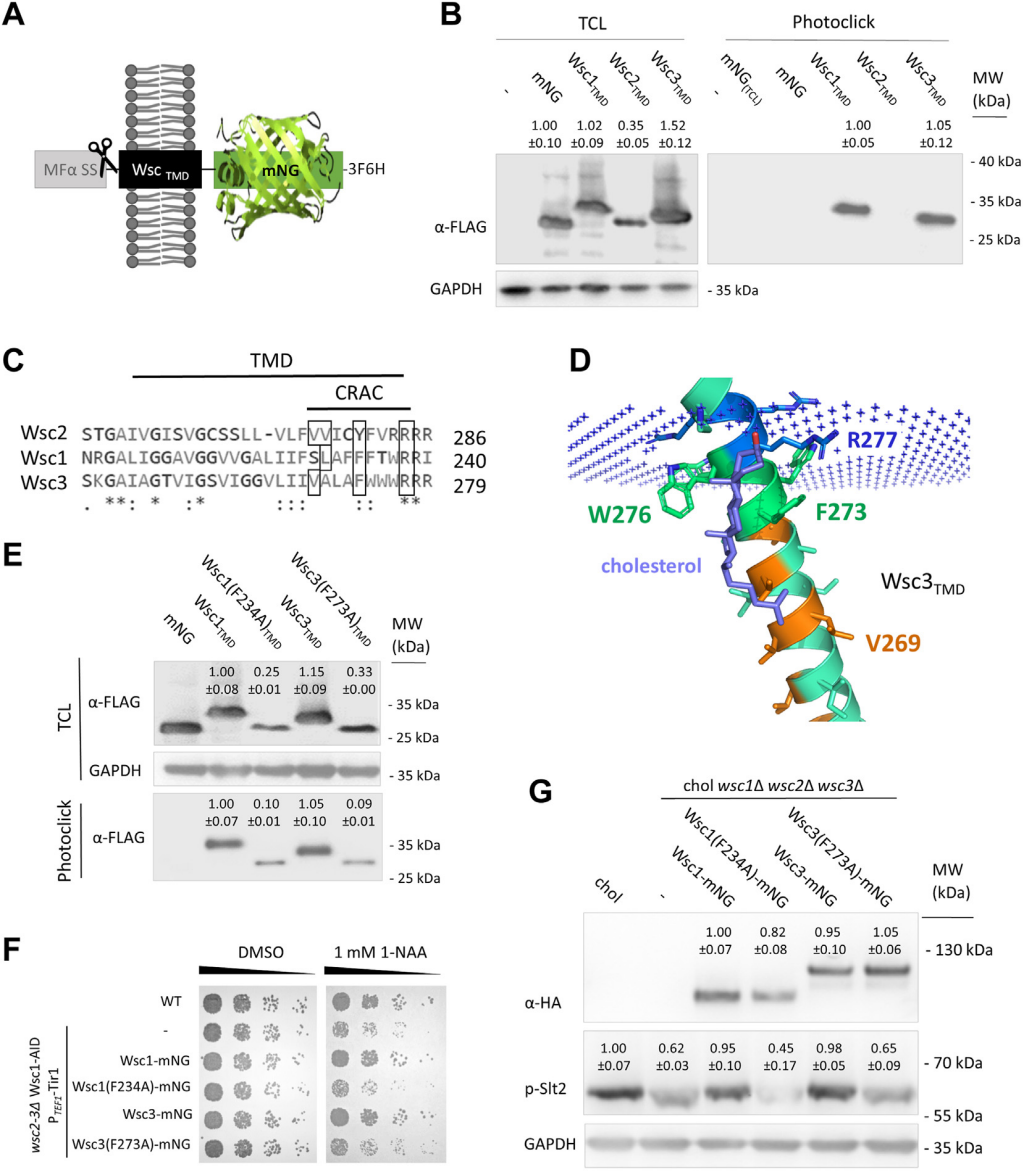
Wsc protein TMDs harbour CRAC domains. A: Scheme of the TMD_Wsc_-mNG probes produced and localized to the cell plasma membrane using the MFα signal sequence. B: For Photoclick assays, the *K. phaffii* strain CBS7435 *his4*Δ, and otherwise isogenic derivatives expressing mNG (yLB227), *WSC1*_TMD_-mNG (yLB200), *WSC2*_TMD_-mNG (yLB201) and *WSC3*_TMD_-mNG (yLB204) from the *TEF1* promoter were cultivated, and prepared for Photoclick assays as described under Materials and Methods. TCL, total cell lysate. MW, marker proteins (kDa). Values above the lanes represent the percentage of relative protein levels (average of three independent experiments with SEM). C: A ClustalW multiple-sequence alignment of *K. phaffii* Wsc1, Wsc2 and Wsc3 revealed high conservation of the respective transmembrane domains, and the presence of possible CRAC motifs. D: Cartoon representation of the Alphafold2 prediction of Wsc3 docked with cholesterol (purple) in its putative orientation in the membrane (blue +). Image was prepared using PyMol (The PyMOL Molecular Graphics System, Version 2.0, Schrödinger, LLC). E: Photoclick assays were performed with microsomal fractions prepared from strains producing mNG (yLB227), Wsc1_TMD_-mNG (yLB200), Wsc1(F234A)_TMD_ -mNG (yLB233), Wsc3_TMD_-mNG (yLB204), and Wsc3(F273A)_TMD_-mNG (yLB234) from the *TEF1* promoter. TCL, total cell lysate. MW, marker proteins (kDa). Values above the lanes represent the percentage of relative protein levels (average of three independent experiments with SEM). F: For spot assays, the *K. phaffii* strain CBS7435 *his4*Δ, a *wsc2-3*Δ double mutant derivative expressing *WSC1*-AID∗ as well as OsTIR1 from the *TEF1* promoter (yLL142), and otherwise isogenic strains expressing *WSC1*-mNG (yLB240), *WSC1*(F234A)-mNG (yLB251), *WSC3*-mNG (yLB243), and *WSC3*(F273A)-mNG (yLB253) were grown in minimal media to late exponential phase, and spotted either on vehicle plates containing 1% DMSO or on plates containing 1 mM 1-NAA as described under Materials and Methods. G: The cholesterol producing strain (MH458), an isogenic derivative carrying deletions of *wsc1*Δ *wsc2*Δ *wsc3*Δ (yLL229), and the same strain expressing *WSC1*-mNG (yLB234), *WSC1*(F234A)-mNG (yLB254), *WSC3*-mNG (yLB237), and *WSC3*(F273A)-mNG (yLB247) were cultivated to middle exponential phase at 28°C, harvested, lysed, and proteins were extracted, resolved by SDS–PAGE, and analyzed by immunoblotting with Phospho-p44/42 MAPK (Erk1/2) (Thr202/Tyr204) antibody and anti-HA antibody, as described under Materials and Methods. Loading control, GAPDH detected on an additional immunoblot where the same samples were loaded using anti-GAPDH antibody. MW, marker proteins (kDa). Values above the lanes represent the percentage of relative protein levels (average of three independent experiments with SEM).

In order to find out, whether Wsc-type sensors directly interact with sterols, we first tried to overproduce Wsc1, Wsc2, and Wsc3. However, overexpression led to extreme flocculation in liquid culture and cells stopped to grow completely after they reached on OD_600_ of 1.0 (supplemental Fig. S3). Seeking an alternative strategy, we constructed Wsc_TMD_-mNG probes, which only contained the transmembrane domains of each Wsc protein and a mNeonGreen-tag (44) as well as a 3xFLAG-(His)_6_-tag (3F6H) attached to the C-terminus protein for detection (Fig. 4A). An improved MFα signal sequence (45) was fused upstream to these chimeras for efficient transport and localization to the plasma membrane. As a control, an mNG-3F6H construct was generated. We verified correct localization by fluorescence microscopy. All three Wsc_TMD_-mNG constructs nicely decorated the plasma membranes of the cells, whereas the mNG control was mostly found in the cytoplasm (supplemental Fig. S4). We generated microsomal fractions enriched in Wsc_TMD_-mNG chimeras and captured the proteins with a photoreactive clickable cholesterol analogue. Immunoblot analysis of the captured proteins identified the presence of Wsc1_TMD_-mNG and Wsc3_TMD_-mNG (Fig. 4B). For the mNG control, we tested both, whole cell extracts as well as microsomal fractions – in none of these cases any interaction with PhotoClick Cholesterol was observed (Fig. 4B), which indicated that the interaction of Wsc_TMD_-mNG and PhotoClick Cholesterol most likely happens at the respective transmembrane domains. By taking a closer look at the transmembrane domains of all three Wsc proteins, we observed possible CRAC motifs (–L/V–(X)(1–5)–Y–(X)(1–5)–R/K–) in all of them (Fig. 4C). The TMD of Wsc2 looks slightly different from those of Wsc1 and Wsc3. First, it is one amino acid shorter, and also, it carries a tyrosin and phenylalanine in the center of the hypothetical CRAC motif. Next, AlphaFold2 predictions were created for these protein domains (Fig. 4D). The predictions of the transmembrane helices of Wsc1 and Wsc2 with AlphaFold2 resulted in models with high predicted IddTs (for the top-ranked prediction 81.23 and 84.98, respectively). For Wsc3, a docking study with cholesterol was performed. The final docking pose, shown in Fig. 4D, represents the highest populated cluster (69 out of 194 samples), and the lowest energy structure. For Wsc1, the same procedure was performed, but no docking pose resembling a productive interaction could be observed. Based on the interaction model we obtained for Wsc3, we chose several amino acids, which—based on their position—may structurally interact with sterols. In Wsc1, L231 (orange), F234 (green), and R238 (blue) (supplemental Fig. S5), and in Wsc3, V269 (orange), F273 (green) and R277 (blue) were identified (Fig. 4D). In an attempt to analyze these interacting sites, we constructed full-length Wsc-mNG and Wsc_TMD_-mNG variants where we exchanged all these amino acids for alanine (supplemental Fig. S6A). However, production could only be verified for very few mutations, namely only for those harboring *WSC1*(F234A) and *WSC3*(F273A). Interestingly, for all different constructs cloned and inserted into the *his4*Δ locus of the *K. phaffii* genome, the same mutations resulted in expression and correct localization to the plasma membrane in the wild type and cholesterol strains (supplemental Fig. S6A), suggesting that the other mutations most probably lead to missfolding and rapid degradation of the respective variants. Proceeding with those expression strains obtained, we conducted different analyses. Expression and correct localization of all constructs was verified by immunoblot analysis (Fig. 4E and supplemental Fig. S6B) and fluorescence microscopy (supplemental Fig. S6C). First, Photoclick assays were performed with microsomal preparation from strains producing Wsc_TMD_-mNG constructs. Even though mutated variants were expressed at reduced levels and exhibited a slightly changed electrophoretic mobility on the gels (Fig. 4E), they correctly localized to the PM of the cells (supplemental Fig. S6C). In the Photoclick experiments, mutated constructs TMD_Wsc1_(F234A), and TMD_Wsc3_(F273A) exhibited slightly reduced interactions with PhotoClick Cholesterol (Fig. 4E). Next, full-length variants were inserted into wild-type *wsc2-3*Δ Wsc1-AID∗ strains and tested for growth complementation on auxin-plates. None of the mutated constructs was able to rescue the growth defect observed when all three Wsc proteins were deleted (Fig. 4F). Full-length constructs were also expressed in the cholesterol *wsc1*Δ *wsc2*Δ *wsc3*Δ triple knockout strain and levels of Slt2-p were monitored. As explained in Materials and Methods, we deliberately used no Slt2-HA strains, since these strains exhibit reduced growth. Also here, while expression of Wsc1-mNG and Wsc3-mNG restored Slt2 overproduction and phosphorylation, mutated variants failed to do so (Fig. 4G).

### Transmembrane domains of integrins interact with PhotoClick cholesterol

**Fig. 5:**
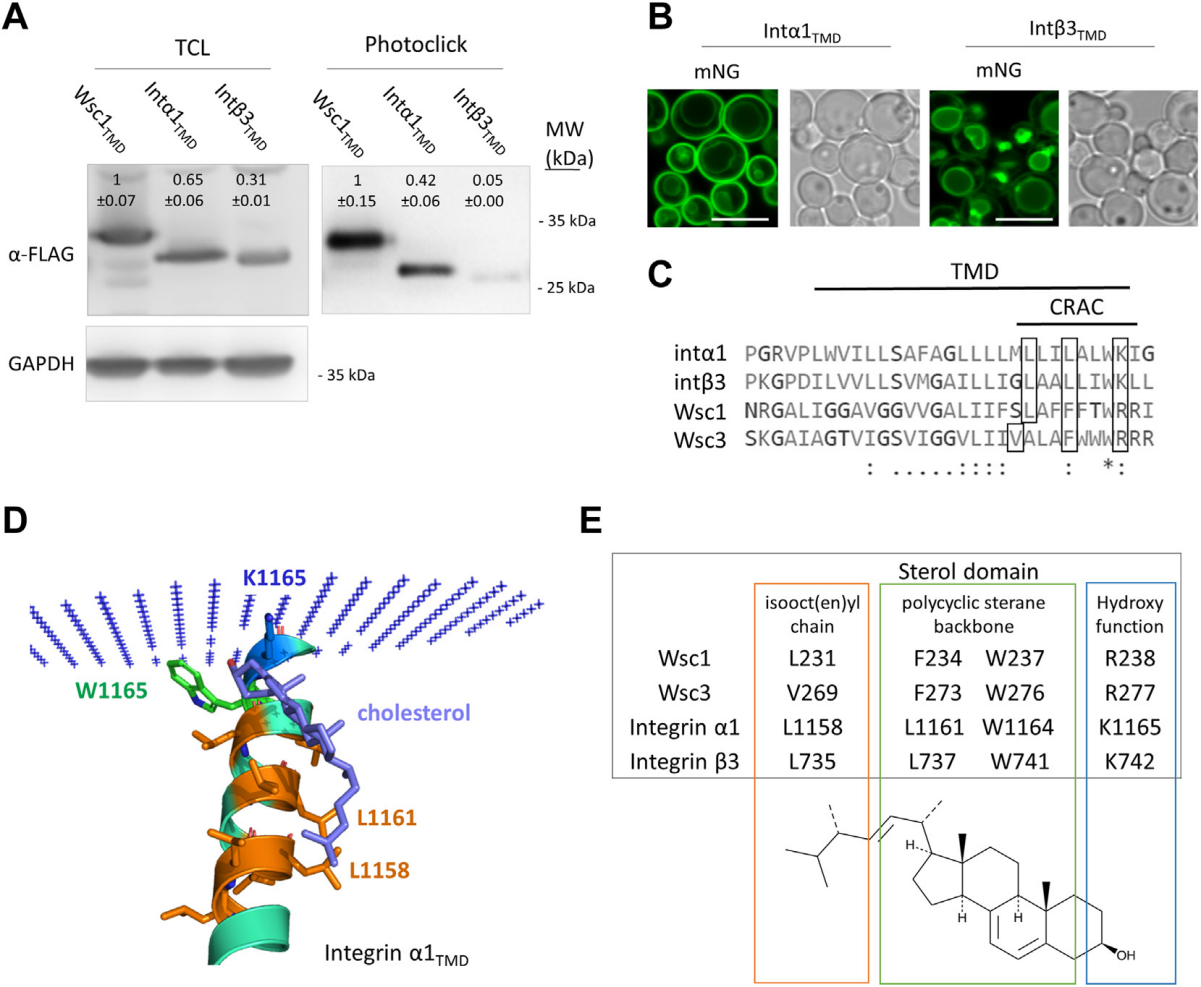
Integrin α1 interacts with PhotoClick Cholesterol. A: For Photoclick assays, the wild type strain expressing *WSC1*_TMD_-mNG (yLB200), *ITGA1*_TMD_-mNG (yPB001), and *ITGB3*_TMD_-mNG (yPB002) were grown to middle exponential phase, harvested, disrupted, microsomal fractions prepared and Photoclick assays performed as described under Materials and Methods. TCL, total cell lysate. MW, marker proteins (kDa). Loading control, GAPDH detected on an additional immunoblot where the same samples were loaded using anti-GAPDH antibody. Values above the lanes represent the percentage of relative protein levels (average of three independent experiments with SEM). B: The same strains as in A were investigated by fluorescence microscopy as described under Materials and Methods. Representative images are shown. Scale bar, 5 μm. C: ClustalW multiple-sequence alignment of *K. phaffii* Wsc1 and Wsc3, and mammalian integrin α1 and integrin β3 revealed high conservation of the respective transmembrane domains. D: Cartoon representation of the Alphafold2 prediction of integrin α1 docked with cholesterol (purple) in its putative orientation in the membrane (blue +). Image was prepared using PyMol (The PyMOL Molecular Graphics System, Version 2.0, Schrödinger, LLC). E: Summary of the potential key players in Wsc proteins and integrins possibly responsible for protein-sterol interaction based on structural modeling.

Integrins are mammalian transmembrane proteins that regulate cellular growth, proliferation, migration, signaling, and cytokine activation, and thereby play important roles in cell proliferation and migration, apoptosis, tissue repair, as well as in all processes critical to inflammation, infection, and angiogenesis. In yeasts, no proteins with sequence similarity to mammalian integrins have been identified yet. However, WSC-type (Wsc1, Wsc2, and Wsc3) and MID-type (Mid2 and Mtl1) mechanosensors have been suggested to resemble integrins in their overall structure and molecular function. (46). Therefore, we chose four different integrin genes, *ITGA1, ITGA2, ITGB1,* and *ITGB3,* took their TMDs, and tried to express them as mNG fusions exactly as described for WscTMD-mNG (Fig. 4A). Only intα1TMD-mNG and intβ3TMD-mNG could be produced in *K. phaffii* (Fig. 5A). Correct localization of these proteins at the plasma membrane was again verified by fluorescence microscopy (Fig. 5B). Intα1TMD-mNG was mostly found at the PM of the cells, while intβ3TMD-mNG mostly accumulated intracellularly, presumably at the periphery of the vacuole. Next, Photoclick assays were performed, and intα1_TMD_-mNG showed interaction with PhotoClick Cholesterol. Intβ3_TMD_-mNG, which did not localize to the PM as robustly, also interacted less strongly with PhotoClick Cholesterol. In the TMDs of integrin α1, integrin β3, Wsc1, and Wsc3, a high sequence identity was identified, and conserved amino acid residues were also found in the potential CRAC domains (Fig. 5C). AlphaFold2 predictions of integrin α1 followed by docking with cholesterol demonstrated the possibility of cholesterol-protein interactions (Fig. 5D). On the basis of experimental and corroborated by modeling results, several amino acid residues were identified in Wsc1, Wsc3, integrin α1, and integrin β3 that can interact with the isooct(en)yl chain, the polycyclic sterane backbone and the hydroxyfunction of sterol molecules (Fig. 5E).

## Discussion

To date, a multitude of mechanisms has been debated of how sterols modulate protein function, from which two major theories emerged. The first is based on the ability of sterols to modify the physical properties of biological membranes by forming liquid-ordered phases. Indeed, plasma membranes of eukaryotic cells contain large amounts of sterols (up to 50 mol%), and the interaction of sterols with the acyl chains of membrane phospholipids promotes tight packing (the so-called “condensing effect” of sterols) (47). As a result, the presence of sterols increases lateral pressure within the membrane, which introduces “packing stress” (48), and this pressure has been proposed as a major mechanism to modulate protein function (48, 49, 50). The second theory, however, proposes that sterols modify protein function by directly binding to the target protein, more specifically to diverse protein-sterol binding motifs. In this study, we discovered that Wsc proteins contain such a binding motif, a so-called CRAC motif, and show that sterol interactions influence correct Wsc protein signaling. In *K. phaffii*, which exclusively harbors Wsc-type sensors, we observed enlarged cell walls and higher levels of Slt2 and p-Slt2 in a cholesterol-producing strain. Induced upregulation of the CWIP, e.g. by short-term exposure (10–45 min) to known cell wall stressors like tunicamycin (51), heat (52), or Congo Red (53) was reported to immediately lead to an upregulation of *SLT2* expression and higher phosphorylation of Slt2 in *S. cerevisiae*. Deletion of all three *WSC* genes, *WSC1*, *WSC2*, and *WSC3* significantly reduced levels of Slt2 and p-Slt2, resulting in reduced cell wall fractions in the cholesterol-producing *K. phaffii* strain, which indicates that these are most likely the key players causing this phenotype. Previously, we have shown that deletion of all three genes coding for Wsc proteins is lethal in a *K. phaffii* wild-type strain (20). The fact that a *wsc1*Δ *wsc2*Δ *wsc3*Δ triple deletion strain is viable when the cell produces cholesterol instead of ergosterol, is remarkable. Slt2 phosphorylation is essential for cell growth in yeast, and the absence of CWI sensors—the most upstream regulators of cell wall signaling—significantly compromises this process (54, 55, 56). Many studies reported crosstalk between different MAP-kinase and stress signaling pathways like the HOG-, CWI-, filamentous growth, and TORC2 signaling pathways in different yeasts (57, 58). The fact that Slt2 phosphorylation only drops to wild-type-like levels in the cholesterol *wsc1*Δ *wsc2*Δ *wsc3*Δ strain indicates that the Wsc proteins are not the only key players involved in causing this phenotype. Instead, other stress response and MAP kinase pathways may be hyperactivated, potentially leading to crosstalk with the CWI pathway and, thereby, rescue Slt2 phosphorylation. A closer analysis of a potential activation of the HOG-, filamentous growth, and TORC2 signaling pathways in the cholesterol strain would be an interesting follow-up experiment.

Fluorescent imaging of mNG-tagged Wsc proteins revealed that Wsc1 and Wsc3 accumulated in the plasma membranes of cholesterol-producing strains (Fig. 3B, C). Previous studies of a cholesterol-producing *S. cerevisiae* strain revealed that correct localization and function of the plasma membrane solute transporters Tat2, Can1 and Pdr12 depends on the presence of ergosterol (59), whereas Can1 and Pdr12 even accumulated in the plasma membranes (60). A similar effect was observed for the yeast pheromone receptor Ste2 in *erg* mutant strains (61). In general, changes in sterol compositions were shown to affect the endocytosis of membrane proteins (25, 61), which could be an explanation, for why Wsc proteins accumulate in the cholesterol-producing strain. Wsc1 was shown to reside in detergent-resistant membrane fractions, i.e. in lipid rafts (8, 62). Lipid rafts are well known for their role in receptor signaling in the plasma membrane and are enriched in sterols and sphingolipids. This raises the question of whether cholesterol production generally blocks endocytosis of Wsc proteins through directly interacting with the protein, or if the impaired endocytic machinery generally affects components of the endocytocytic pathway, as shown in many cases in the past (63).

PhotoClick Cholesterol is globally used to map protein-ergosterol interaction (64, 65). In our case, the construction of non-functional Wsc-protein TMD-mNG chimeras enabled efficient, high-level expression of the target sequences of interest. This approach also limited the possibility of unspecific binding of PhotoClick Cholesterol to the extensive extracellular or intracellular portions of Wsc proteins. PhotoClick Cholesterol is efficiently bound to Wsc1_TMD_- and Wsc3 _TMD_ -mNG, which indicates close interaction of the respective transmembrane domains with the sterol compound. When we mutated the central phenylalanine of the CRAC motif, protein-sterol interactions were reduced. Similar results were also published by other groups (66, 67), whereas, in other cases, the central aromatic amino acid was shown not to be involved in sterol binding at all (68). It is important to note that the overall structure of PhotoClick Cholesterol is somewhat different from cholesterol. First, it lacks the double bond in the four hydrocarbon ring structure between positions 5 and 6 due to the presence of the UV-activatable, cross-linking diazirine group at position 6. Second, it carries an alkyne group for incorporation of reporter and affinity tags through click-chemistry instead of the isobutyl group at position 24. Assuming that close structural interaction of sterols is important for correct function, our findings suggest that the structural differences of cholesterol and ergosterol (Fig. 2A) are the main reason for upregulated Wsc protein signaling. Overall, this seems barely surprising considering the fact that a single alpha-helical transmembrane domain is exclusively responsible for transmitting any signal detected by the extracellular portions to the intracellular parts of Wsc sensors. Hence, any destabilization caused by structural differences of a tightly interacting sterol could have a huge impact on the signaling and functionality of Wsc proteins.

Finally, we found that integrin and Wsc protein TMDs are highly conserved and that integrin transmembrane domains also interact with PhotoClick Cholesterol, even though they are missing the typical aromatic amino acid in the core of the hypothetical CRAC domain. Interestingly, the innermost, tryptophan of the transmembrane domains is the only residue that is fully conserved between Wsc proteins and integrins, and docking experiments exhibited close structural proximity of these residues with sterol molecules (Figs. 4D and 5D). It even appears as if the arginine or lysin and the tryptophan of Wsc proteins and integrins, respectively, engulf the hydroxyfunction and the first hydrophobic ring of the four hydrocarbon ring structures like a forefinger and a thumb.

In the literature, there are several hints concerning the influence of cholesterol on integrins. First, integrins, which also contain only one single alpha-helical transmembrane domain, preferentially cluster in cholesterol-enriched lipid rafts (69). Second, integrins showed reduced microclustering (69), higher mobility (70) and changes in sequestration (69) in membranes containing lower concentrations of cholesterol. In these studies, the unison hypothesis was that changes in lipid packing and bilayer thickness majorly influence integrin mobility, activity and localization. While we cannot rule out that differences in the packaging of biological membranes containing cholesterol instead of ergosterol influence Wsc protein signaling, our results indicate that Wsc proteins require close structural interaction with their natural sterol species for correct function and that similar interactions might be required for integrins.

## Data Availability

This study includes no data deposited in external repositories.

## Supplemental data

This article contains supplemental data (20, 60, 71, 72, 73).

## Conflict of interest

The authors declare that they have no known competing financial interests or personal relationships that could have appeared to influence the work reported in this paper.

## Supplemental Data

Supplemental Table S2

pAEA424_pPpHyg-Cas9-HIS4.ape

pAEA426_pPpKC3 Wsc1-mNG.ape.ape

pAEA429_pPpHyg-Cas9-SLT2.ape

pAEA462_pPpKC2_his4-PWsc3-Wsc3-mNG.gb

pLB142_pPpKC3 Wsc2-mNG-3HA.gb

pLB143_pPpKC3 Wsc3-mNG-3HA.gb

pLB216_pPpKC2_his4-TEF1-MFa-wsc1TMD-mNG-3F6H.gb

pLB217_pPpKC2_his4-TEF1-MFa-Wsc2TMD-mNG-3F6H.gb

pLB218_pPpKC2_his4-TEF1-MFa-Wsc3TMD-mNG-3F6H.gb

pLB221_pPpKC2_his4-TEF1-mNG-3F6H.gb

pLB222_A179_HYGdual_wsc1ko.gbk.ape

pLB224_pPphis4-TEF1-MFa-wsc1TMD-F234A-mNG-3F6H.gb

pLB225_pPphis4-TEF1-MFa-Wsc3TMD-F273A-mNG-3F6H.gb

pLB233_pPpKC2_his4-PWsc1-Wsc1(F234A)-mNG.gb

pLB234_pPpKC2_his4-PWsc3(F273A)-Wsc3-mNG.gb

pLB235_pPpKC2_his4-PWsc2-Wsc2-mNG.gb

pLL016_pPpKC2_his4-PWsc1-Wsc1-mNG.gb

pPB001_pPpKC2_his4-TEF1-MFa-integrin alpha-1 TMD-mNG-3F6H.gb

pPB002_pPpKC2_his4-TEF1-MFa-integrin beta 3 TMD-mNG-3F6H.gb
