# Supplementary material for: Sterol interactions influence the function of Wsc sensors
Source: J Lipid Res. 2023 Nov 2;64(12):100466. doi: 10.1016/j.jlr.2023.100466 (PMC10722382; doi:10.1016/j.jlr.2023.100466)
Supplement: Supplemental Figures and Tables [file mmc1.docx]

**Supplemental Figures**

**
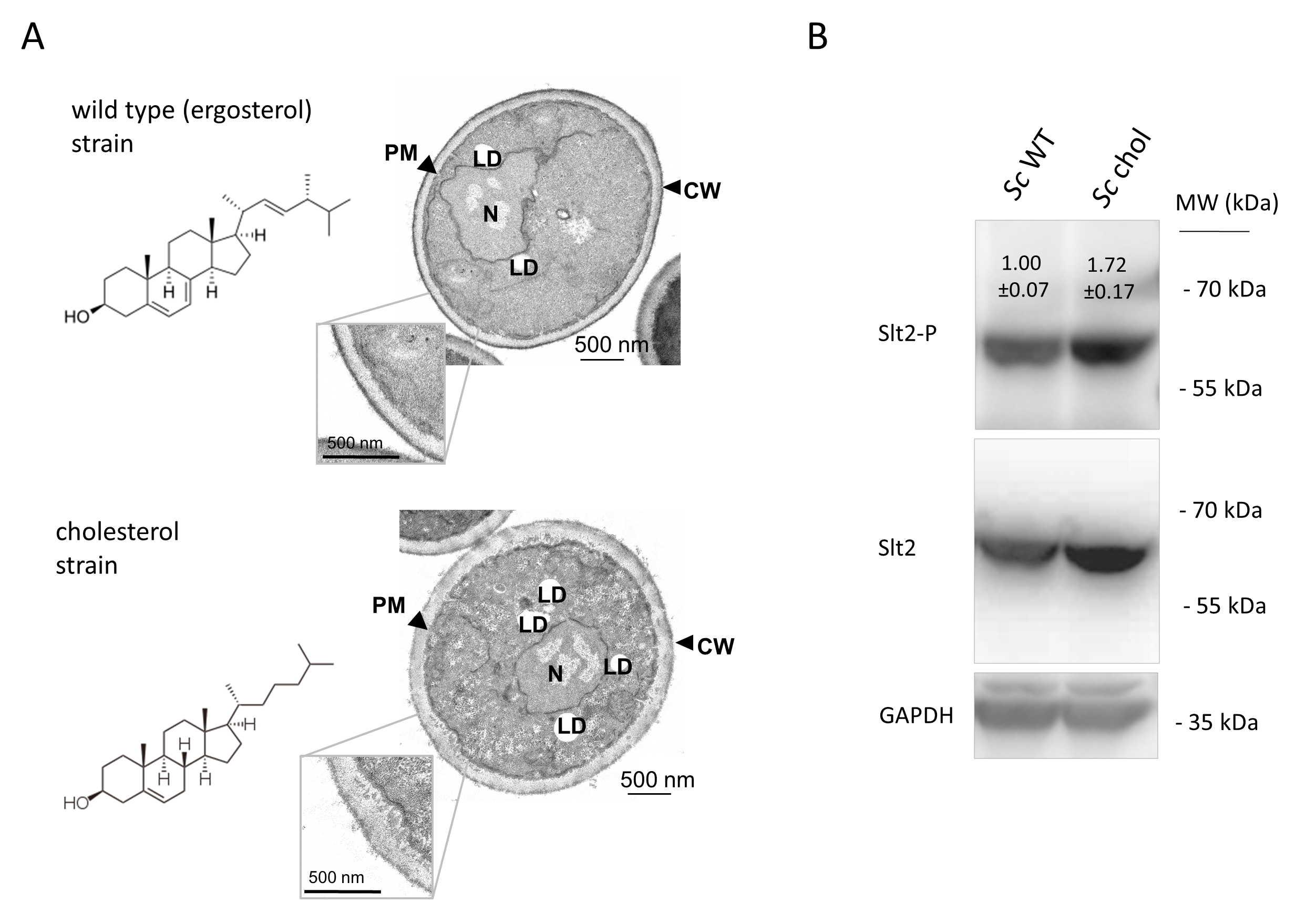
Supplemental Figure S1 - Cell wall biosynthesis is upregulated when ergosterol is exchanged for cholesterol in *S. cerevisiae*.** A) *S. cerevisiae* strain RH2881 and an isogenic derivative harbouring gene insertions *erg5*::DHRC7 and *erg6*::DHCR24 for cholesterol production (RH6829), were cultivated to middle exponential phase and imaged by TEM as described under Materials and Methods. Scale bar, 500 nm. PM: plasma membrane, CW: cell wall, V: vacuole, N: nucleus, LD: lipid droplets. B) The same strains as in A were cultivated to middle exponential phase at 28°C, harvested, lysed, and proteins were extracted, resolved by SDS–PAGE, and analyzed by immunoblotting with Phospho-p44/42 MAPK (Erk1/2) (Thr202/Tyr204) antibody and anti-Mpk1 antibody, as described under Materials and Methods. Loading control, GAPDH detected on the same immunoblots using anti-GAPDH antibody. MW, marker proteins (kDa). Values above the lanes represent the percentage of relative protein levels (average of three independent experiments with SEM).

**
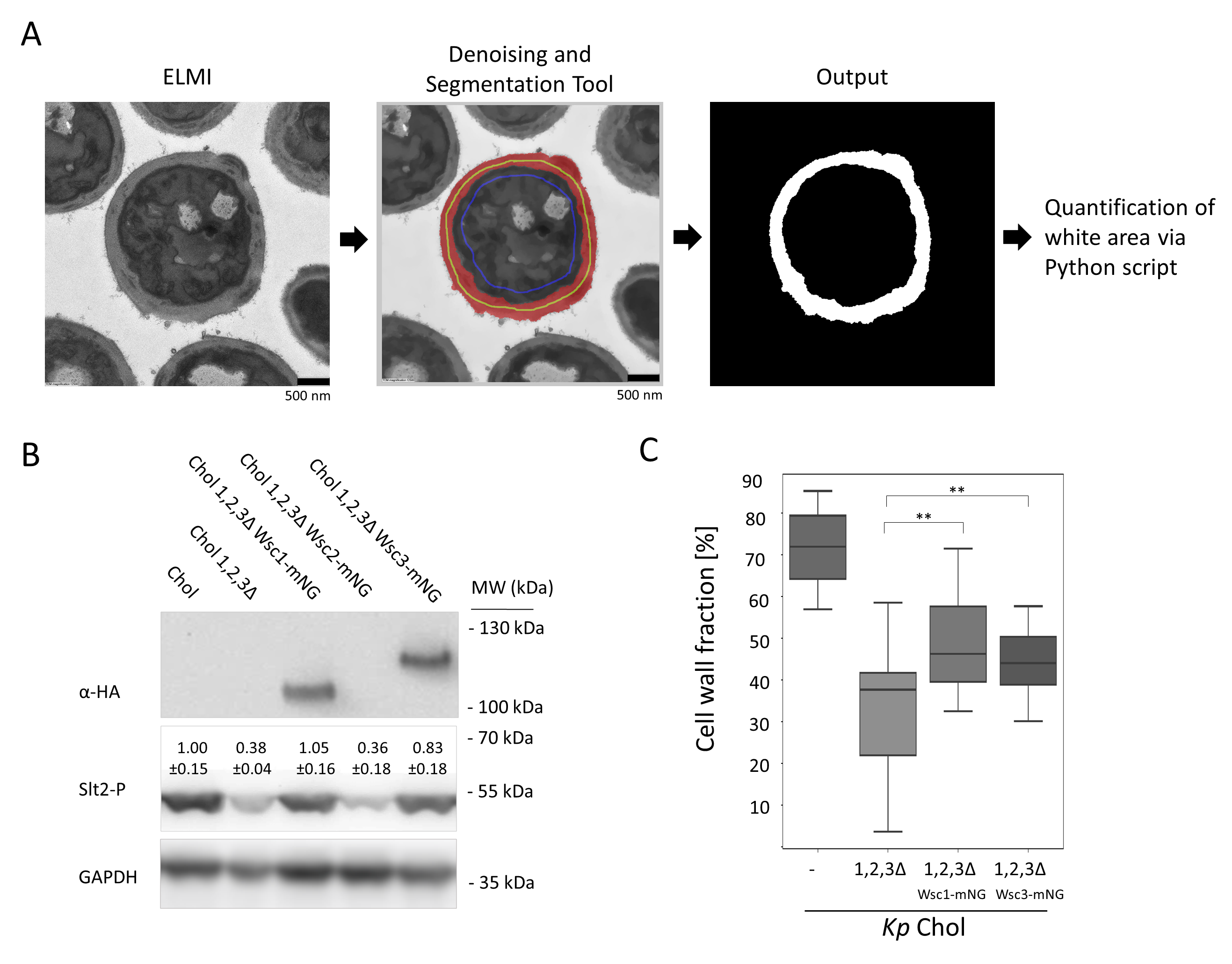
 Supplemental Figure S2 - Wsc proteins are responsible for cell wall enlargement in cholesterol producing *K. phaffii* cells.** A) Cell wall quantification is exemplified for a cholesterol producing *K. phaffii* cell (MH458). Cells were cultivated and harvested, and imaged with TEM as described under Materials and Methods. Images were processed using a ‘Denoising and Segmentation Tool’, which allows for segmenting of cell wall areas and provides black white images as output files. Scale bar, 500 nm. B) A cholesterol producing strain (MH458), an isogenic derivative that harbours deletions of *wsc1*∆, *wsc2*∆ and *wsc3*∆ (yLB229) and the same strain expressing copies of *WSC1* (yLB234), *WSC2* (yLB255) and *WSC*3 (yLB237) from its endogenous promoters as integrated cassette from the *his4*∆ locus were cultivated to middle exponential phase at 28°C, harvested, lysed, and proteins were extracted, resolved by SDS–PAGE, and analyzed by immunoblotting with anti-HA, Phospho-p44/42 MAPK (Erk1/2) (Thr202/Tyr204) antibody and anti-Mpk1 antibody, as described under Materials and Methods. Loading control, GAPDH detected on the same immunoblots using anti-GAPDH antibody. MW, marker proteins (kDa). Values above the lanes represent the percentage of relative protein levels (average of three independent experiments with SEM). C) The same strains as in B (except for yLB255) were grown to middle exponential phase and imaged by fluorescence microscopy. Cell wall areas from electron microscopy images (*n* = 15 per strain) were quantified using a ‘Denoising and Segmentation Tool’ as described in Materials and Methods, and results plotted in box-and-whisker format.


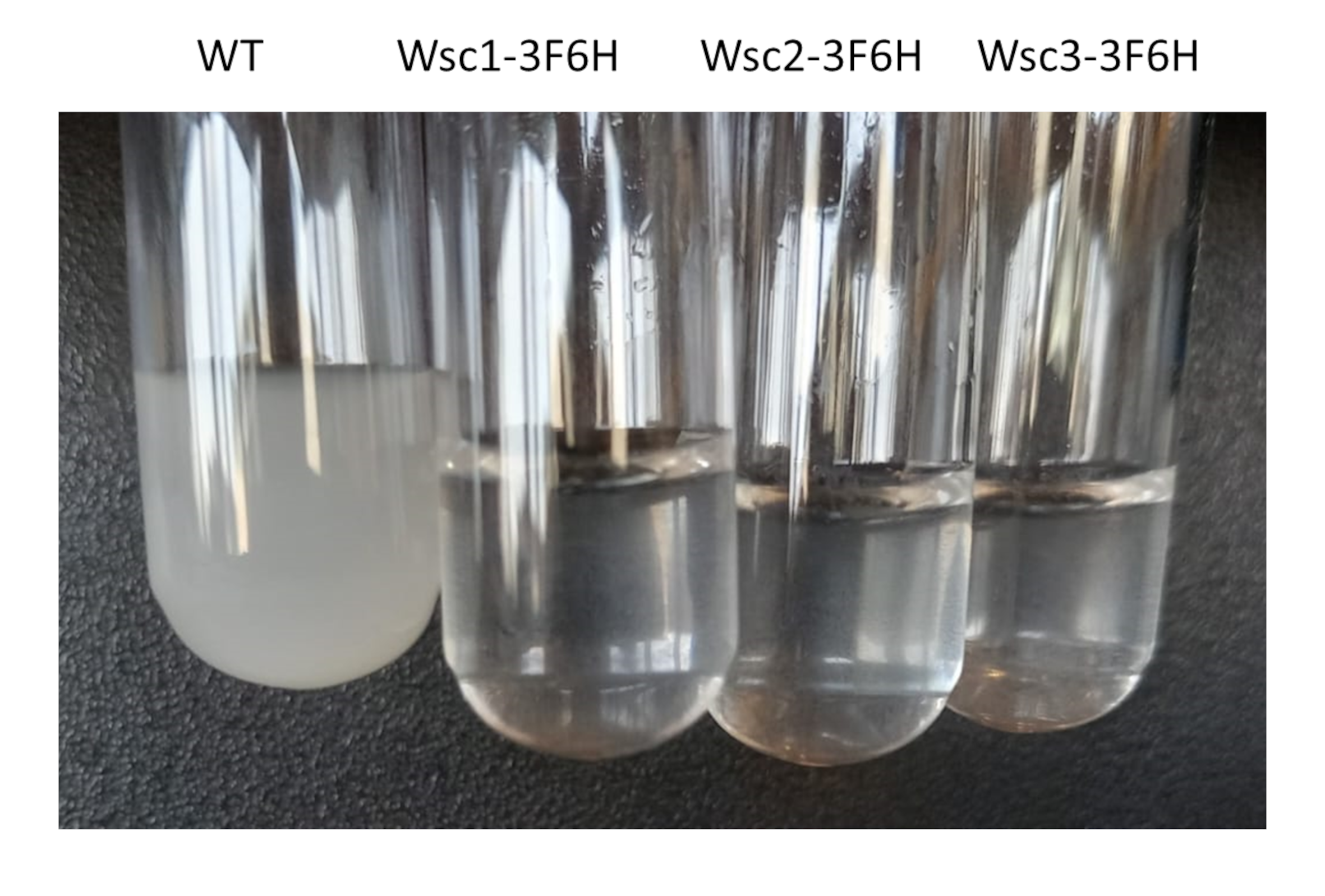
 **Supplemental Figure S3 - *K. phaffii* strains overproducing Wsc proteins exhibit extreme flocculation.** A wild type *K. phaffii* strain (CBS 7435) was used for overexpressing *WSC1*-3FLAG-(His)_6_ (yLB200), *WSC1*-3FLAG-(His)_6_ (yLB201), and *WSC1*-3FLAG-(His)_6_ (yLB204) from the *TEF2* promoter. Cells were cultivated in 2 mL of MD media at 28°C for 24 h.


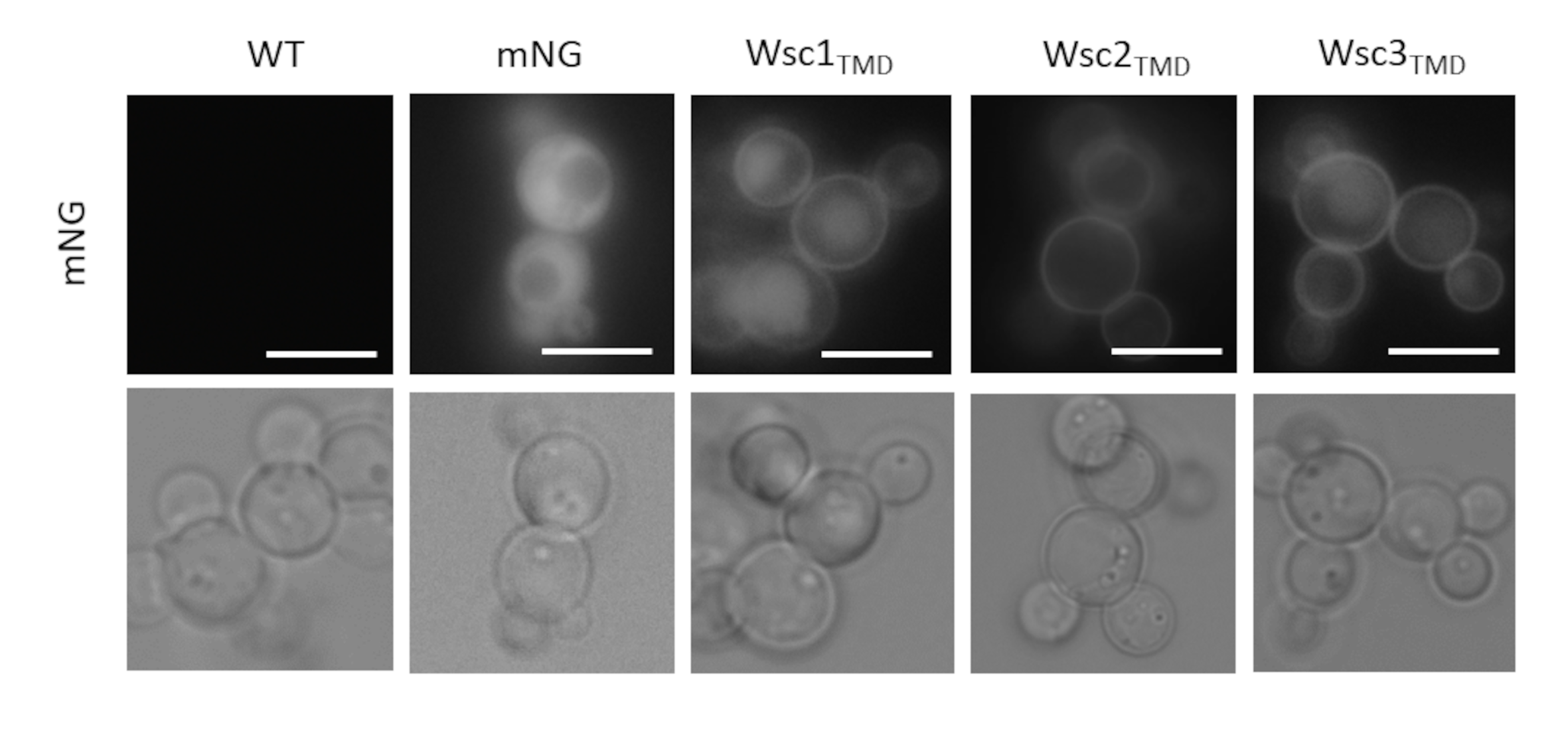


**Supplemental Figure S4 - Wsc_TMD_-mNG constructs localize correctly to the plasma membrane.** Strains producing mNG (yLB227), Wsc1_TMD_-mNG (yLB200), Wsc1(F234A)_TMD_-mNG (yLB233), Wsc3_TMD_-mNG (yLB204), and Wsc3(F273A)_TMD_-mNG (yLB234) from the *TEF1* promoter were grown to middle exponential phase and examined by fluorescence microscopy. Representative images are shown. Scale bar, 5 μm.


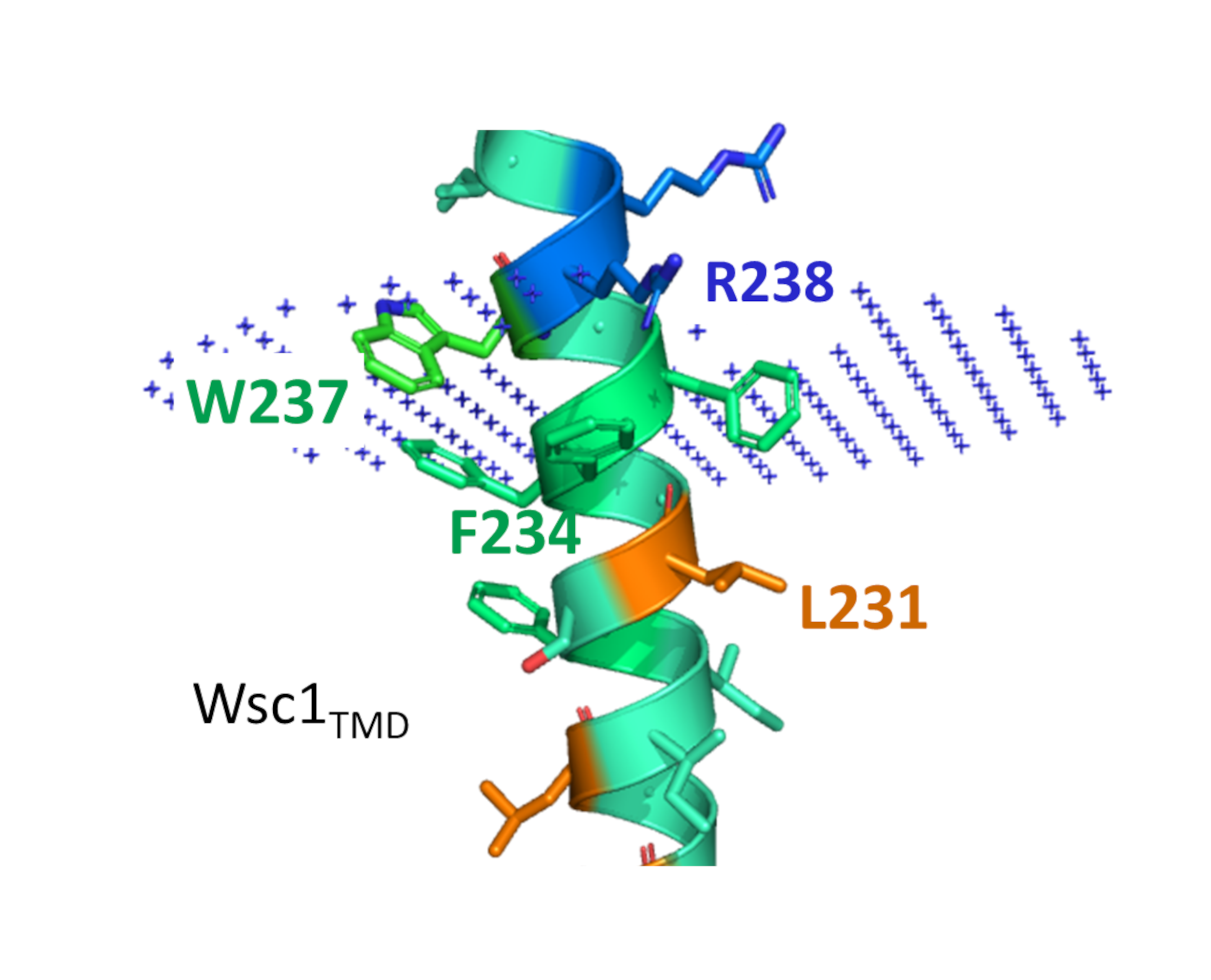


**Supplemental Figure S5 - Cartoon representation of the Alphafold2 prediction of Wsc3 and its putative orientation in a membrane (blue +).** The Figure was prepared using PyMol (The PyMOL Molecular Graphics System, Version 2.0, Schrödinger, LLC).

**
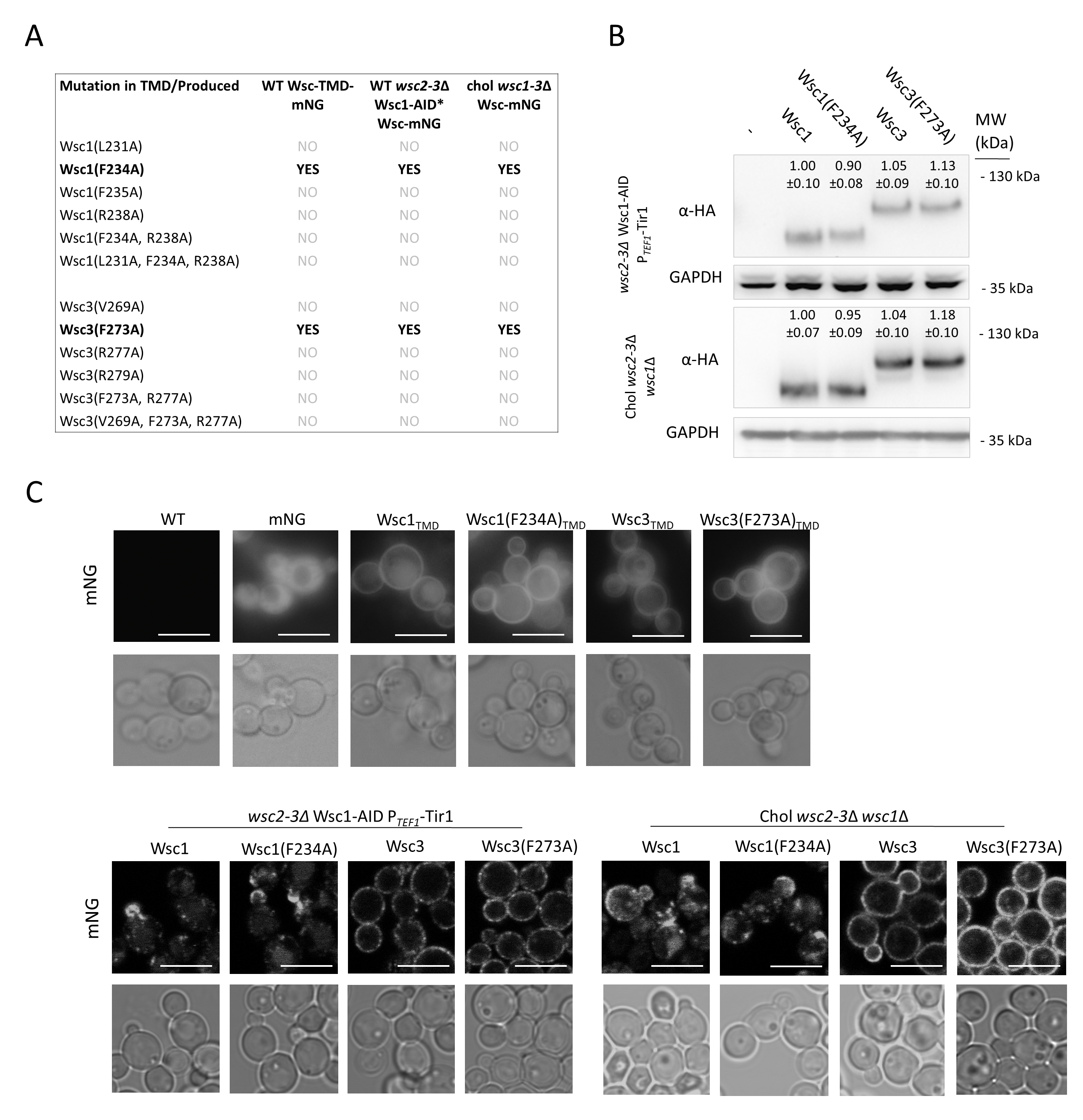
 Supplemental Figure S6 - Mutational analysis of transmembrane domains of Wsc1 and Wsc3.** A) Overview of different constructs cloned for expression in wild type and cholesterol producing strains. Expression of mutated constructs was only confirmed for highlighted constructs and strains. B) One way to confirm expression was immunoblot analysis. Therefore, the *K. phaffii* strain CBS7435 *his4*∆ *wsc2-3*∆ double mutant expressing *WSC1*-AID* as well as OsTIR1 from the *TEF1* promoter (yLL142), and otherwise isogenic strains expressing *WSC1*-mNG (yLB240), *WSC1*(F234A)-mNG (yLB251), *WSC3*-mNG (yLB243), and *WSC3*(F273A)-mNG (yLB253); and a cholesterol producing strain that harbours deletions of *wsc1*∆, *wsc2*∆ and *wsc3*∆ (yLB229) and the same strain expressing copies of *WSC1*-mNG (yLB234), *WSC1*(F234A)-mNG (yLB254), *WSC3*-mNG (yLB237) and *WSC3*(F273A)-mNG (yLB247) from its endogenous promoters were cultivated to middle exponential phase at 28°C, harvested, lysed, and proteins were extracted, resolved by SDS–PAGE, and analyzed by immunoblotting with anti-HA antibody, as described under Materials and Methods. Loading control, GAPDH detected on the same immunoblots using anti-GAPDH antibody. MW, marker proteins (kDa). C) The same strains as in B, as well as strains producing mNG (yLB227), Wsc1_TMD_-mNG (yLB200), Wsc1(F234A)_TMD_-mNG (yLB233), Wsc3_TMD_-mNG (yLB204), and Wsc3(F273A)_TMD_-mNG (yLB234) from the *TEF1* promoter were grown to middle exponential phase and examined by fluorescence microscopy. Representative images are shown. Scale bar, 5 μm.

**Supplemental Tables**

**Supplemental Table S1: Strategies and plasmids applied for strain generation**

| CRISPR/Cas9 strategies | | | |
| --- | --- | --- | --- |
| Purpose | sgRNA targeting site and PAM sequence (Cas9 plasmid) | Preparation of repair cassette | Yeast strain(s) generated |
| 3HA-tagging of *SLT2* | TGAATTAGAGTCCCAAATTTTGG * (pAEA429, pPpHyg-Cas9-*SLT2*) | *SLT2*-HA repair cassette* generated by PCR | yAEA361, yAEA384, yAEA366, yAEA367, yAEA390, yAEA377. yAEA369, yAEA379 |
| *WSC1-*mNG-3HA integrated into *his4*∆ locus | TTATCAGTGAGTCAGTCATCAGG (pAEA424, pPpHyg-Cas9-*HIS4*) | pLL016 cut with *Smi*I and 3360 bp band purified | yLB240, yLB234 |
| *WSC2-*mNG-3HA integrated into *his4*∆ locus | TTATCAGTGAGTCAGTCATCAGG (pAEA424, pPpHyg-Cas9-*HIS4*) | pL235 cut with *Smi*I and 3723 bp band purified | yLB241 |
| *WSC3-*mNG-3HA integrated into *his4*∆ locus | TTATCAGTGAGTCAGTCATCAGG (pAEA424, pPpHyg-Cas9-*HIS4*) | pAEA462 cut with *Smi*I and 3518 bp band purified | yLB243, yLB237 |
| mNG-3F6H integrated into *his4*∆ locus | TTATCAGTGAGTCAGTCATCAGG (pAEA424, pPpHyg-Cas9-*HIS4*) | pLB221 cut with *Smi*I and 2514 bp band purified | yLB227 |
| *WSC1*_TMD_-3F6H integrated into *his4*∆ locus | TTATCAGTGAGTCAGTCATCAGG (pAEA424, pPpHyg-Cas9-*HIS4*) | pLB216 cut with *Smi*I and 2844 bp band purified | yLB200 |
| *WSC2*_TMD_-3F6H integrated into *his4*∆ locus | TTATCAGTGAGTCAGTCATCAGG (pAEA424, pPpHyg-Cas9-*HIS4*) | pLB217 cut with *Smi*I and 2904 bp band purified | yLB201 |
| *WSC3*_TMD_-3F6H integrated into *his4*∆ locus | TTATCAGTGAGTCAGTCATCAGG (pAEA424, pPpHyg-Cas9-*HIS4*) | pLB218 cut with *Smi*I and 2847 bp band purified | yLB204 |
| *WSC1*(F234A)_TMD_-3F6H integrated into *his4*∆ locus | TTATCAGTGAGTCAGTCATCAGG (pAEA424, pPpHyg-Cas9-*HIS4*) | pLB224 cut with *Smi*I and 2844 bp band purified | yLB233 |
| *WSC3*(F273A)_TMD_-3F6H integrated into *his4*∆ locus | TTATCAGTGAGTCAGTCATCAGG (pAEA424, pPpHyg-Cas9-*HIS4*) | pLB225 cut with *Smi*I and 2847 bp band purified | yLB234 |
| *WSC1*(F234A)*-*mNG-3HA integrated into *his4*∆ locus | TTATCAGTGAGTCAGTCATCAGG (pAEA424, pPpHyg-Cas9-*HIS4*) | pLB233 cut with *Smi*I and 3360 bp band purified | yLB251, yLB254 |
| *WSC3*(F273A)*-*mNG-3HA integrated into *his4*∆ locus | TTATCAGTGAGTCAGTCATCAGG (pAEA424, pPpHyg-Cas9-*HIS4*) | pLB234 cut with *Smi*I and 3518 bp band purified | yLB253, yLB247 |
| Frameshift mutation in *WSC1* | GTCTTCTGCCTCTTCATTAGCGG (pLB222, pPpHyg-Cas9-*WSC1*) | - | yLB229 |
| *ITGA1*_TMD_-3F6H integrated into *his4*∆ locus | TTATCAGTGAGTCAGTCATCAGG (pAEA424, pPpHyg-Cas9-*HIS4*) | pPB001 cut with *Smi*I and 2898 bp band purfied | yPB001 |
| *ITGB3*_TMD_-3F6H integrated into *his4*∆ locus | TTATCAGTGAGTCAGTCATCAGG (pAEA424, pPpHyg-Cas9-*HIS4*) | pPB002 cut with *Smi*I and 2904 bp band purfied | yPB002 |
| Conventional integration of cassettes | | | |
| Purpose | Generation of cassette | Selection | Used to generate yeast strain(s) |
| mNG-tagging of *WSC1* in the genome | Cut pAEA426 with *Smi*I, purify 5179 bp band | MD-his plates | yAEA345, yAEA348 |
| mNG-tagging of *WSC2* in the genome | Cut pLB142 with *Smi*I, purify 7948 bp band | MD-his plates | yLB130, yLB136 |
| mNG-tagging of *WSC3* in the genome | Cut pLB143 with *Smi*I, purify 7942 bp band | MD-his plates | yLB133, yLB139 |

*CATCTTCCTAGACCTCATGAATTAGAGTCCCAAATTTACCCATACGATGTTCCTGACTATGCGTTAGATCTAGAACATGAATTACAATACGGATTGGATC

**Supplemental Table S2: Yeast strains used in this study**

| *K. phaffii* strains | | |
| --- | --- | --- |
| Strain ID | Genotype | Reference |
| CBS7435 | his4Δ | (Näätsaari *et al*, 2012) |
| MH458 | CBS7435 *his4*Δ *erg5*::*GAPDH* _prom_-DHCR7 *erg6*:: *GAPDH* _prom_-DHCR24 | (Hirz *et al*, 2013) |
| yAEA361 | CBS7435 *his4*Δ *SLT2-*3HA | This study |
| yAEA384 | CBS7435 *his4*Δ *erg5*::DHCR7 *erg6*::DHCR24 *SLT2-*3HA | This study |
| yAEA366 | CBS7435 *his4*Δ *erg5*::DHCR7 *erg6*::DHCR24 *wsc1::HIS4 SLT2-*3HA | This study |
| yAEA387 | CBS7435 *his4*Δ *erg5*::DHCR7 *erg6*::DHCR24 *wsc2::HIS4 SLT2-*3HA | This study |
| yAEA367 | CBS7435 *his4*Δ *erg5*::DHCR7 *erg6*::DHCR24 *wsc3::HIS4 SLT2-*3HA | This study |
| yAEA390 | CBS7435 *his4*Δ *erg5*::DHCR7 *erg6*::DHCR24 *wsc2::HIS4 wsc1::HYG SLT2-*3HA | This study |
| yAEA377 | CBS7435 *his4*Δ *erg5*::DHCR7 *erg6*::DHCR24 *wsc1::HYG wsc3::HIS4 SLT2-*3HA | This study |
| yAEA369 | CBS7435 *his4*Δ *erg5*::DHCR7 *erg6*::DHCR24 *wsc2-3::HIS4 SLT2-*3HA | This study |
| yAEA379 | CBS7435 *his4*Δ *erg5*::DHCR7 *erg6*::DHCR24 *wsc2-3::HIS4 wsc1::HYG SLT2-*3HA | This study |
| yLL142 | CBS7435 his4Δ wsc2-3::HIS4 WSC1_prom_::WSC1-AID∗-3HA TEF2_prom_-TIR1-FLAG | (Lehmayer *et al*, 2022) |
| yLB240 | CBS7435 his4Δ wsc2-3::HIS4 WSC1_prom_::WSC1-AID∗-3HA TEF2_prom_-TIR1-FLAG  *his4::WSC1*_prom_-*WSC1*-mNG-3HA | This study |
| yLB241 | CBS7435 his4Δ wsc2-3::HIS4 WSC1_prom_::WSC1-AID∗-3HA TEF2_prom_-TIR1-FLAG  *his4::WSC2*_prom_-*WSC2*-mNG-3HA | This study |
| yLB243 | CBS7435 his4Δ wsc2-3::HIS4 WSC1_prom_::WSC1-AID∗-3HA TEF2_prom_-TIR1-FLAG  *his4::WSC3*_prom_-*WSC3*-mNG-3HA | This study |
| yAEA345 | CBS7435 *his4*Δ *WSC1*_prom_-*WSC1*-mNG-3HA | This study |
| yAEA348 | CBS7435 *his4*Δ *erg5*::DHCR7 *erg6*::DHCR24  *WSC1*_prom_-*WSC1*-mNG-3HA | This study |
| yLB130 | CBS7435 *his4*Δ *WSC2*_prom_-*WSC2*-mNG-3HA | This study |
| yLB136 | CBS7435 *his4*Δ *erg5*::DHCR7 *erg6*::DHCR24  *WSC2*_prom_-*WSC2*-mNG-3HA | This study |
| yLB133 | CBS7435 *his4*Δ *WSC3*_prom_-*WSC3*-mNG-3HA | This study |
| yLB139 | CBS7435 *his4*Δ *erg5*::DHCR7 *erg6*::DHCR24  *WSC3*_prom_-*WSC3*-mNG-3HA | This study |
| yLB227 | CBS7435 *his4*Δ *his4::TEF2_prom_*-mNG-3FLAG-(HIS)_6_ | This study |
| yLB200 | CBS7435 *his4*Δ *his4::TEF2_prom_*-*WSC1*_TMD_-mNG-3FLAG-(HIS)_6_ | This study |
| yLB201 | CBS7435 *his4*Δ *his4::TEF2_prom_*-*WSC2* _TMD_ -mNG-3FLAG-(HIS)_6_ | This study |
| yLB204 | CBS7435 *his4*Δ *his4::TEF2_prom_*-*WSC3*_TMD_-mNG-3FLAG-(HIS)_6_ | This study |
| yLB233 | CBS7435 *his4*Δ *his4::TEF2_prom_*-*WSC1*(F234A)_TMD_-mNG-3FLAG-(HIS)_6_ | This study |
| yLB234 | CBS7435 *his4*Δ *his4::TEF2_prom_*-*WSC3***(F273A)**_TMD_-mNG-3FLAG-(HIS)_6_ | This study |
| yLB251 | CBS7435 his4Δ wsc2-3::HIS4 WSC1_prom_::WSC1-AID∗-3HA TEF2_prom_-TIR1-FLAG  *his4::WSC1*_prom_-*WSC1*(F234A)-mNG-3HA | This study |
| yLB253 | CBS7435 his4Δ wsc2-3::HIS4 WSC1_prom_::WSC1-AID∗-3HA TEF2_prom_-TIR1-FLAG  *his4::WSC3*_prom_-*WSC3***(F273A)-**mNG-3HA | This study |
| yLB229 | CBS7435 *his4*Δ *erg5*::DHCR7 *erg6*::DHCR24 *wsc2-3::HIS4 wsc1*Δ | This study |
| yLB234 | CBS7435 *his4*Δ *erg5*::DHCR7 *erg6*::DHCR24 *wsc2-3::HIS4 wsc1*Δ *his4::WSC1*_prom_-*WSC1*-mNG-3HA | This study |
| yLB254 | CBS7435 *his4*Δ *erg5*::DHCR7 *erg6*::DHCR24 *wsc2-3::HIS4 wsc1*Δ *his4::WSC1*_prom_-*WSC1(F234A)*-mNG-3HA | This study |
| yLB237 | CBS7435 *his4*Δ *erg5*::DHCR7 *erg6*::DHCR24 *wsc2-3::HIS4 wsc1*Δ *his4::WSC3*_prom_-*WSC3*-mNG-3HA | This study |
| yLB247 | CBS7435 *his4*Δ *erg5*::DHCR7 *erg6*::DHCR24 *wsc2-3::HIS4 wsc1*Δ *his4::WSC3*_prom_-*WSC3(F273A)*-mNG-3HA | This study |
| yLB255 | CBS7435 *his4*Δ *erg5*::DHCR7 *erg6*::DHCR24 *wsc2-3::HIS4 wsc1*Δ *his4::WSC2*_prom_-*WSC2*-mNG-3HA | This study |
| yPB001 | CBS7435 *his4*Δ *his4::TEF2_prom_*-*ITGA1*_TMD_-mNG-3FLAG-(HIS)_6_ | This study |
| yPB002 | CBS7435 *his4*Δ *his4::TEF2_prom_*-*ITGB3* _TMD_ -mNG-3FLAG-(HIS)_6_ | This study |
| *S. cerevisiae* strains | | |
| Strain ID | Genotype | Reference |
| RH2881 | W303-1A MAT**a** *ura3*Δ *leu2*Δ *his3*Δ *trp1*Δ *can1*Δ *bar1*Δ | (Schorling *et al*, 2001) |
| RH6829 | W303-1A MAT**a** *ura3*Δ *leu2*Δ *his3*Δ *trp1*Δ *can1*Δ *bar1*Δ *erg5*::*HIS3*-*TDH3* _prom_-DHCR24 *erg6*::*TRP1*-*TDH3* _prom_-DHCR7 | (Souza *et al*, 2011) |
