# Supplementary material for: Sterol interactions influence the function of Wsc sensors
Source: J Lipid Res. 2023 Nov 2;64(12):100466. doi: 10.1016/j.jlr.2023.100466 (PMC10722382; doi:10.1016/j.jlr.2023.100466)
Supplement: Supplemental Table S1 [file mmc2.docx]

**Supplemental Table S1: Strategies and plasmids applied for strain generation**

| CRISPR/Cas9 strategies | | | | |
| --- | --- | --- | --- | --- |
| Purpose | sgRNA targeting site and PAM sequence (Cas9 plasmid) | Preparation of repair cassette | Yeast strain(s) generated |  |
| 3HA-tagging of *SLT2* | TGAATTAGAGTCCCAAATTTTGG * (pAEA429, pPpHyg-Cas9-*SLT2*) | *SLT2*-HA repair cassette* generated by PCR | yAEA361, yAEA384, yAEA366, yAEA367, yAEA390, yAEA377. yAEA369, yAEA379 |  |
| *WSC1-*mNG-3HA integrated into *his4*∆ locus | TTATCAGTGAGTCAGTCATCAGG (pAEA424, pPpHyg-Cas9-*HIS4*) | pLL016 cut with *Smi*I and 3360 bp band purified | yLB240, yLB234 |  |
| *WSC2-*mNG-3HA integrated into *his4*∆ locus | TTATCAGTGAGTCAGTCATCAGG (pAEA424, pPpHyg-Cas9-*HIS4*) | pL235 cut with *Smi*I and 3723 bp band purified | yLB241 |  |
| *WSC3-*mNG-3HA integrated into *his4*∆ locus | TTATCAGTGAGTCAGTCATCAGG (pAEA424, pPpHyg-Cas9-*HIS4*) | pAEA462 cut with *Smi*I and 3518 bp band purified | yLB243, yLB237 |  |
| mNG-3F6H integrated into *his4*∆ locus | TTATCAGTGAGTCAGTCATCAGG (pAEA424, pPpHyg-Cas9-*HIS4*) | pLB221 cut with *Smi*I and 2514 bp band purified | yLB227 |  |
| *WSC1*_TMD_-3F6H integrated into *his4*∆ locus | TTATCAGTGAGTCAGTCATCAGG (pAEA424, pPpHyg-Cas9-*HIS4*) | pLB216 cut with *Smi*I and 2844 bp band purified | yLB200 |  |
| *WSC2*_TMD_-3F6H integrated into *his4*∆ locus | TTATCAGTGAGTCAGTCATCAGG (pAEA424, pPpHyg-Cas9-*HIS4*) | pLB217 cut with *Smi*I and 2904 bp band purified | yLB201 |  |
| *WSC3*_TMD_-3F6H integrated into *his4*∆ locus | TTATCAGTGAGTCAGTCATCAGG (pAEA424, pPpHyg-Cas9-*HIS4*) | pLB218 cut with *Smi*I and 2847 bp band purified | yLB204 |  |
| *WSC1*(F234A)_TMD_-3F6H integrated into *his4*∆ locus | TTATCAGTGAGTCAGTCATCAGG (pAEA424, pPpHyg-Cas9-*HIS4*) | pLB224 cut with *Smi*I and 2844 bp band purified | yLB233 |  |
| *WSC3*(F273A)_TMD_-3F6H integrated into *his4*∆ locus | TTATCAGTGAGTCAGTCATCAGG (pAEA424, pPpHyg-Cas9-*HIS4*) | pLB225 cut with *Smi*I and 2847 bp band purified | yLB234 |  |
| *WSC1*(F234A)*-*mNG-3HA integrated into *his4*∆ locus | TTATCAGTGAGTCAGTCATCAGG (pAEA424, pPpHyg-Cas9-*HIS4*) | pLB233 cut with *Smi*I and 3360 bp band purified | yLB251, yLB254 |  |
| *WSC3*(F273A)*-*mNG-3HA integrated into *his4*∆ locus | TTATCAGTGAGTCAGTCATCAGG (pAEA424, pPpHyg-Cas9-*HIS4*) | pLB234 cut with *Smi*I and 3518 bp band purified | yLB253, yLB247 |  |
| Frameshift mutation in *WSC1* | GTCTTCTGCCTCTTCATTAGCGG (pLB222, pPpHyg-Cas9-*WSC1*) | - | yLB229 |  |
| *ITGA1*_TMD_-3F6H integrated into *his4*∆ locus | TTATCAGTGAGTCAGTCATCAGG (pAEA424, pPpHyg-Cas9-*HIS4*) | pPB001 cut with *Smi*I and 2898 bp band purfied | yPB001 |  |
| *ITGB3*_TMD_-3F6H integrated into *his4*∆ locus | TTATCAGTGAGTCAGTCATCAGG (pAEA424, pPpHyg-Cas9-*HIS4*) | pPB002 cut with *Smi*I and 2904 bp band purfied | yPB002 |  |
| Conventional integration of cassettes | | | | |
| Purpose | Generation of cassette | Selection | Used to generate yeast strain(s) |  |
| mNG-tagging of *WSC1* in the genome | Cut pAEA426 with *Smi*I, purify 5179 bp band | MD-his plates | yAEA345, yAEA348 |  |
| mNG-tagging of *WSC2* in the genome | Cut pLB142 with *Smi*I, purify 7948 bp band | MD-his plates | yLB130, yLB136 |  |
| mNG-tagging of *WSC3* in the genome | Cut pLB143 with *Smi*I, purify 7942 bp band | MD-his plates | yLB133, yLB139 |  |

*CATCTTCCTAGACCTCATGAATTAGAGTCCCAAATTTACCCATACGATGTTCCTGACTATGCGTTAGATCTAGAACATGAATTACAATACGGATTGGATC
