# Supplementary material for: Sterol interactions influence the function of Wsc sensors
Source: J Lipid Res. 2023 Nov 2;64(12):100466. doi: 10.1016/j.jlr.2023.100466 (PMC10722382; doi:10.1016/j.jlr.2023.100466)
Supplement: Supplemental Table S2 [file mmc3.docx]

**Supplemental Table S2: Yeast strains used in this study**

| *K. phaffii* strains | | |
| --- | --- | --- |
| Strain ID | Genotype | Reference |
| CBS7435 | his4Δ | (Näätsaari *et al*, 2012) |
| MH458 | CBS7435 *his4*Δ *erg5*::*GAPDH* _prom_-DHCR7 *erg6*:: *GAPDH* _prom_-DHCR24 | (Hirz *et al*, 2013) |
| yAEA361 | CBS7435 *his4*Δ *SLT2-*3HA | This study |
| yAEA384 | CBS7435 *his4*Δ *erg5*::DHCR7 *erg6*::DHCR24 *SLT2-*3HA | This study |
| yAEA366 | CBS7435 *his4*Δ *erg5*::DHCR7 *erg6*::DHCR24 *wsc1::HIS4 SLT2-*3HA | This study |
| yAEA387 | CBS7435 *his4*Δ *erg5*::DHCR7 *erg6*::DHCR24 *wsc2::HIS4 SLT2-*3HA | This study |
| yAEA367 | CBS7435 *his4*Δ *erg5*::DHCR7 *erg6*::DHCR24 *wsc3::HIS4 SLT2-*3HA | This study |
| yAEA390 | CBS7435 *his4*Δ *erg5*::DHCR7 *erg6*::DHCR24 *wsc2::HIS4 wsc1::HYG SLT2-*3HA | This study |
| yAEA377 | CBS7435 *his4*Δ *erg5*::DHCR7 *erg6*::DHCR24 *wsc1::HYG wsc3::HIS4 SLT2-*3HA | This study |
| yAEA369 | CBS7435 *his4*Δ *erg5*::DHCR7 *erg6*::DHCR24 *wsc2-3::HIS4 SLT2-*3HA | This study |
| yAEA379 | CBS7435 *his4*Δ *erg5*::DHCR7 *erg6*::DHCR24 *wsc2-3::HIS4 wsc1::HYG SLT2-*3HA | This study |
| yLL142 | CBS7435 his4Δ wsc2-3::HIS4 WSC1_prom_::WSC1-AID∗-3HA TEF2_prom_-TIR1-FLAG | (Lehmayer *et al*, 2022) |
| yLB240 | CBS7435 his4Δ wsc2-3::HIS4 WSC1_prom_::WSC1-AID∗-3HA TEF2_prom_-TIR1-FLAG  *his4::WSC1*_prom_-*WSC1*-mNG-3HA | This study |
| yLB241 | CBS7435 his4Δ wsc2-3::HIS4 WSC1_prom_::WSC1-AID∗-3HA TEF2_prom_-TIR1-FLAG  *his4::WSC2*_prom_-*WSC2*-mNG-3HA | This study |
| yLB243 | CBS7435 his4Δ wsc2-3::HIS4 WSC1_prom_::WSC1-AID∗-3HA TEF2_prom_-TIR1-FLAG  *his4::WSC3*_prom_-*WSC3*-mNG-3HA | This study |
| yAEA345 | CBS7435 *his4*Δ *WSC1*_prom_-*WSC1*-mNG-3HA | This study |
| yAEA348 | CBS7435 *his4*Δ *erg5*::DHCR7 *erg6*::DHCR24  *WSC1*_prom_-*WSC1*-mNG-3HA | This study |
| yLB130 | CBS7435 *his4*Δ *WSC2*_prom_-*WSC2*-mNG-3HA | This study |
| yLB136 | CBS7435 *his4*Δ *erg5*::DHCR7 *erg6*::DHCR24  *WSC2*_prom_-*WSC2*-mNG-3HA | This study |
| yLB133 | CBS7435 *his4*Δ *WSC3*_prom_-*WSC3*-mNG-3HA | This study |
| yLB139 | CBS7435 *his4*Δ *erg5*::DHCR7 *erg6*::DHCR24  *WSC3*_prom_-*WSC3*-mNG-3HA | This study |
| yLB227 | CBS7435 *his4*Δ *his4::TEF2_prom_*-mNG-3FLAG-(HIS)_6_ | This study |
| yLB200 | CBS7435 *his4*Δ *his4::TEF2_prom_*-*WSC1*_TMD_-mNG-3FLAG-(HIS)_6_ | This study |
| yLB201 | CBS7435 *his4*Δ *his4::TEF2_prom_*-*WSC2* _TMD_ -mNG-3FLAG-(HIS)_6_ | This study |
| yLB204 | CBS7435 *his4*Δ *his4::TEF2_prom_*-*WSC3*_TMD_-mNG-3FLAG-(HIS)_6_ | This study |
| yLB233 | CBS7435 *his4*Δ *his4::TEF2_prom_*-*WSC1*(F234A)_TMD_-mNG-3FLAG-(HIS)_6_ | This study |
| yLB234 | CBS7435 *his4*Δ *his4::TEF2_prom_*-*WSC3***(F273A)**_TMD_-mNG-3FLAG-(HIS)_6_ | This study |
| yLB251 | CBS7435 his4Δ wsc2-3::HIS4 WSC1_prom_::WSC1-AID∗-3HA TEF2_prom_-TIR1-FLAG  *his4::WSC1*_prom_-*WSC1*(F234A)-mNG-3HA | This study |
| yLB253 | CBS7435 his4Δ wsc2-3::HIS4 WSC1_prom_::WSC1-AID∗-3HA TEF2_prom_-TIR1-FLAG  *his4::WSC3*_prom_-*WSC3***(F273A)-**mNG-3HA | This study |
| yLB229 | CBS7435 *his4*Δ *erg5*::DHCR7 *erg6*::DHCR24 *wsc2-3::HIS4 wsc1*Δ | This study |
| yLB234 | CBS7435 *his4*Δ *erg5*::DHCR7 *erg6*::DHCR24 *wsc2-3::HIS4 wsc1*Δ *his4::WSC1*_prom_-*WSC1*-mNG-3HA | This study |
| yLB254 | CBS7435 *his4*Δ *erg5*::DHCR7 *erg6*::DHCR24 *wsc2-3::HIS4 wsc1*Δ *his4::WSC1*_prom_-*WSC1(F234A)*-mNG-3HA | This study |
| yLB237 | CBS7435 *his4*Δ *erg5*::DHCR7 *erg6*::DHCR24 *wsc2-3::HIS4 wsc1*Δ *his4::WSC3*_prom_-*WSC3*-mNG-3HA | This study |
| yLB247 | CBS7435 *his4*Δ *erg5*::DHCR7 *erg6*::DHCR24 *wsc2-3::HIS4 wsc1*Δ *his4::WSC3*_prom_-*WSC3(F273A)*-mNG-3HA | This study |
| yLB255 | CBS7435 *his4*Δ *erg5*::DHCR7 *erg6*::DHCR24 *wsc2-3::HIS4 wsc1*Δ *his4::WSC2*_prom_-*WSC2*-mNG-3HA | This study |
| yPB001 | CBS7435 *his4*Δ *his4::TEF2_prom_*-*ITGA1*_TMD_-mNG-3FLAG-(HIS)_6_ | This study |
| yPB002 | CBS7435 *his4*Δ *his4::TEF2_prom_*-*ITGB3* _TMD_ -mNG-3FLAG-(HIS)_6_ | This study |
| *S. cerevisiae* strains | | |
| Strain ID | Genotype | Reference |
| RH2881 | W303-1A MAT**a** *ura3*Δ *leu2*Δ *his3*Δ *trp1*Δ *can1*Δ *bar1*Δ | (Schorling *et al*, 2001) |
| RH6829 | W303-1A MAT**a** *ura3*Δ *leu2*Δ *his3*Δ *trp1*Δ *can1*Δ *bar1*Δ *erg5*::*HIS3*-*TDH3* _prom_-DHCR24 *erg6*::*TRP1*-*TDH3* _prom_-DHCR7 | (Souza *et al*, 2011) |
